# Establishment of a clinical diagnostic model for gouty arthritis based on the serum biochemical profile: A case-control study

**DOI:** 10.1097/MD.0000000000025542

**Published:** 2021-04-23

**Authors:** Shang Lyu, Ruowen Ding, Shilin Yang, Wanyuan Chen, Yi Rao, Hui OuYang, Peng Liu, Yulin Feng

**Affiliations:** a National Pharmaceutical Engineering Center for Solid Preparation in Chinese Herbal Medicine, Jiangxi University of Traditional Chinese Medicine; b Department of Pathology, Zhejiang Provincial People's Hospital, People's Hospital of Hangzhou Medical College, Hangzhou; c State Key Laboratory of Innovative Drug and Efficient Energy-Saving Pharmaceutical Equipment, Nanchang, China.

**Keywords:** clinical diagnostic model, disease progression, gouty arthritis, multiple logistic regression analysis, serum biochemical profile

## Abstract

The disease progression of gouty arthritis (GA) is relatively clear, with the 4 stages of hyperuricemia (HUA), acute gouty arthritis (AGA), gouty arthritis during the intermittent period (GIP), and chronic gouty arthritis (CGA). This paper attempts to construct a clinical diagnostic model based on blood routine test data, in order to avoid the need for bursa fluid examination and other tedious steps, and at the same time to predict the development direction of GA.

Serum samples from 579 subjects were collected within 3 years in this study and were divided into a training set (n = 379) and validation set (n = 200). After a series of multivariate statistical analyses, the serum biochemical profile was obtained, which could effectively distinguish different stages of GA. A clinical diagnosis model based on the biochemical index of the training set was established to maximize the probability of the stage as a diagnosis, and the serum biochemical data from 200 patients were used for validation.

The total area under the curve (AUC) of the clinical diagnostic model was 0.9534, and the AUCs of the 5 models were 0.9814 (Control), 0.9288 (HUA), 0.9752 (AGA), 0.9056 (GIP), and 0.9759 (CGA). The kappa coefficient of the clinical diagnostic model was 0.80.

This clinical diagnostic model could be applied clinically and in research to improve the accuracy of the identification of the different stages of GA. Meanwhile, the serum biochemical profile revealed by this study could be used to assist the clinical diagnosis and prediction of GA.

## 1 Introduction

Gouty arthritis (GA) is a kind of chronic disease caused by purine metabolic disorders, and its progression is relatively clear, which can be roughly divided into the following 4 stages: hyperuricemia (HUA), acute gouty arthritis (AGA), gouty arthritis during the intermittent period (GIP), and chronic gouty arthritis (CGA). When the uric acid (UA) level in blood is above 7 mg/L or 420 μmol/L (HUA), monosodium urate (MSU) crystals might accumulate in the joint capsule, bursa, cartilage, bone, or other periarticular tissues, which can stimulate the synovial membrane of the joint and produce pathological reactions, such as synovial vasodilation, as well as increased permeability and leukocyte exudation (AGA). However, some patients with HUA do not progress to AGA and only show excessive serum UA levels.^[1]^ Statistics show that up to 30% of AGA patients had normal UA values.^[2,3]^ Therefore, a high UA level in blood is a necessary and insufficient condition for AGA. Clinically, AGA is most frequently encountered in the major joints, especially in the first metatarsophalangeal joint, ankle, and foot joints. Other complications include chronic renal injury, ureteral calculi, and arthritis malformation.^[4,5]^ Long-term intermittent repeated episodes of AGA would lead to the deposition of uratoma and eventually evolve into CGA, and there would be an asymptomatic interval (GIP) from the evolution of AGA to CGA (see Figure S1, Supplemental Content, which demonstrates the abridged general view of the evolution of GA).

According to statistics, the incidence rate of GA is increasing every year globally (about 1%–2%, 2018), especially in developing countries.^[6,7]^ With the deepening of research in recent years, GA has been gradually defined as an autoimmune inflammatory disease.^[8]^ Some preliminary research conclusions on the pathogenesis of AGA and CGA have been made by researchers at present. The pathogenesis of AGA might be related to the activation of Toll-like receptors, the NLRP3 inflammasome, or the P2X7 receptor,^[9]^ while CGA might be related to the stimulation of the generation of extracellular neutrophil traps^[10]^ or endoplasmic reticulum stress responses.^[11]^ Some scholars also analyzed the metabolites of gout in HUA patients and healthy volunteers by nuclear magnetic resonance (NMR)^[12]^ and ion chromatography-mass spectrometry (IC-MS),^[13]^ and thus obtained the relevant biomarkers of gout and HUA including oxalic acid, l-homocysteic acid, lipids, and amino acids, which could help in developing lab tests for GA.

The clinical diagnosis of GA has been generally based on joint swelling,^[14]^ CT findings,^[15]^ smear test results,^[16]^ and patient descriptions, which involve uncertainties (MSU crystal smear of HUA has positive results).^[17]^ At present, there are different treatment methods for GA patients at different stages, such as diet control, drug treatment, and surgical treatment. However, in the absence of clear diagnostic markers, there is still a lack of methods to prevent the occurrence of GA and predict the evolution trend of GA. Therefore, the white blood cell (WBC) count, C-reactive protein (CRP) level, UA level, blood urea nitrogen (BUN) level, creatinine (Cre) level, hemoglobin (Hem) level, erythrocyte sedimentation rate (ESR), high/low-density lipoprotein (HDL/LDL) level, total cholesterol (TC) level, triglyceride (TG) level, demographics (age, body mass index [BMI], and sex), and living habit (smoking and alcohol drinking habit) have been considered as candidate risk factors of the progression of GA in this research. For most patients, the symptoms of GA and rheumatoid arthritis (RA) are similar, so it is easy to ignore the condition and delay the optimal treatment time. As a consequence, we also distinguished the 2 by the difference in biochemical indicators. Furthermore, principal component analysis (PCA), orthogonal partial least squares discrimination analysis (OPLS-DA), non-repetitive one-way analysis of variance (ANOVA),^[18]^ correlation analysis,^[19]^ and multiple logistic regression analysis^[20]^ were used to screen the important indicators affecting GA in each stage, and to distinguish GA and RA. Finally, multiple logistic regression analysis was used to establish a clinical diagnosis model,^[21]^ so as to improve the success rate of clinical diagnosis and prediction (see Figure S2, Supplemental Content, which demonstrates the overview of the study design).

## 2 Methods

### 2.1 Study design and group forming criterion

This study was implemented from November 30, 2017 to November 10, 2019, at Zhejiang Provincial People's Hospital (ZPPH), the largest comprehensive first-class hospital in Zhejiang province of China. All participants were categorized as healthy controls, HUA patients, AGA patients, GIP patients, and CGA patients based on the level of serum UA and the definition of GA as follows (based on the 2015 ACR-EULAR Gout Classification Criteria)^[22]^:

(1) healthy controls were considered as those with a serum UA level of 150 to 416 μmol/L in men or 80 to 357 μmol/L in women and without GA or other types of arthritis, such as RA, osteoarthritis, and psoriatic arthritis.
(2) HUA patients were considered as those with a serum UA level of >416 μmol/L in men or >357 μmol/L in women.
(3) AGA patients were considered as those with persistent swelling and intense pain in the peripheral joints or bursae, with symptom peak within 24 hours and resolution within 14 days.
(4) GIP patients were considered as those in whom it had been over 4 weeks since the last AGA attack and who had not received UA lowering therapy (ULT).
(5) CGA patients were considered as those with joint swelling, pressing pain, deformity, dysfunction, and hypodermic tophus.
(6) RA patients were considered as those with multi-joint swelling, disease duration over 6 weeks and rheumatoid factor (RF) >20. In the HUA and AGA groups, all synovial fluid samples were examined using polarized-light microscopy to ensure the presence of MSU crystals.

### 2.2 Measurement method and instruments

In addition to HUA, the onset of GA was usually associated with age, BMI, sex, smoking, and alcohol drinking habit.^[3]^ Therefore, uric acid level, WBC, CRP, urea nitrogen, creatinine, hemoglobin, erythrocyte sedimentation rate (ESR), LDL, HDL, total cholesterol, triglyceride, demographic data, living habits, comorbidities (tumors and cardiovascular diseases), disease duration, and medical situations were collected through questionnaires and case history. Serum UA levels and other blood biochemical indexes of participants in each group were determined by the CHEMIX-180 automatic biochemistry analyzer (Sysmex Corp., Kobe, Japan). Each participant was visited, and serum was collected only once during the study period.

### 2.3 Study population

A total of 579 serum specimens were collected from ZPPH, including 379 in the training set (80 healthy volunteers [21.1%], 62 patients with HUA [16.4%], 69 patients with AGA [18.2%], 74 patients with GIP [19.5%], 62 patients with CGA [16.4%], and 32 patients with RA [8.4%]). As well as 200 in the validation set (80 healthy volunteers [40.0%], 30 patients with HUA [15.0%], 30 patients with AGA [15.0%], 30 patients with GIP [15.0%], and 30 patients with CGA [15.0%]).

About 70% of the patients were inpatients and another 30% were outpatients. The acute episodes of AGA lasted no <3 days, and the course of CGA was >11 years. Among the 207 patients with GA (mean age 49.23 ± 17.90 years), 188 were men (90.8%) and 19 (9.2%) were women. Compared with controls, GA patients had a higher average BMI (25.88 ± 3.01 vs 22.9 ± 2.7 kg/m^2^; *P* = .01) and UA level (420.81 ± 116.86 vs 158.2 ± 89.0 μmol/L; *P* = .001). Meanwhile, the proportions of individuals with smoking and alcohol drinking habits were higher among GA patients than among healthy volunteers (smoking: 34.4% vs 55.8%; drinking: 25.0% vs 46.3%).

### 2.4 Statistical analysis methods

**Figure 1:**
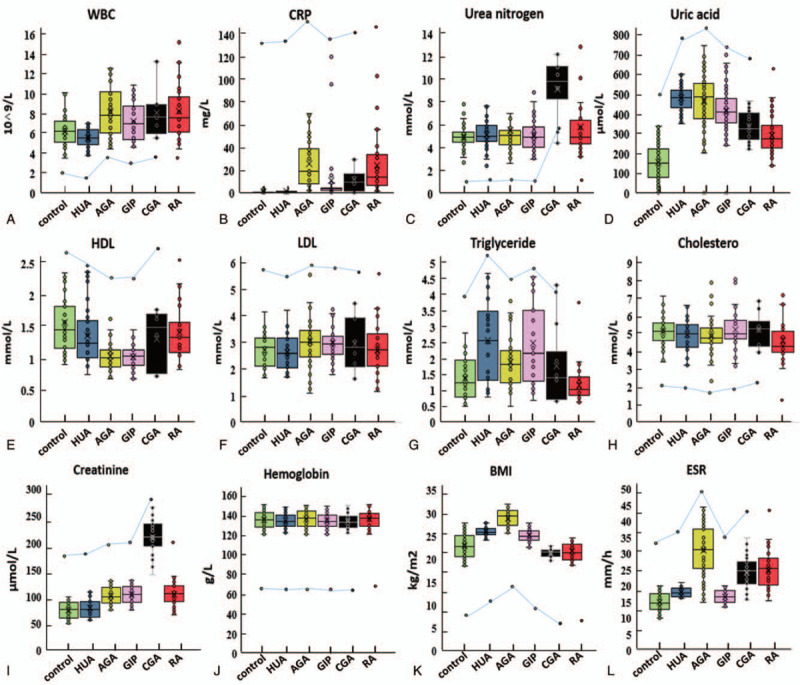
The 8 selected blood biochemical indexes for the identification of the 5 GA stages and RA. A–L Box plots show the blood concentrations of the 8 indexes (left *y*-axis), including the WBC count, CRP level, uric acid level, urea nitrogen level, HDL/LDL level, cholesterol level, triglyceride level, creatinine level, hemoglobin level, BMI, and ESR across all 5 GA stages and RA. In the box plot, the median is expressed by the center line, 75% is represented by the upper bound of the box, 25% is expressed by the lower bound of the box, minimum is expressed by the lower whisker, and maximum is expressed by the upper whisker. Dot plots and lines show the trend of indexes across all 5 GA stages and RA. BMI = body mass index, CRP = C reactive protein, ESR = erythrocyte sedimentation rate; GA = gouty arthritis, HDL/LDL = high/low-density lipoprotein, RA = rheumatoid arthritis, WBC = white blood cell.

Statistical descriptions of other biochemical indicators in all serum specimens are provided in Table 1, and the trends of biochemical indicators in the 5 disease progressions are indicated by box plots (Fig. 1). The difference in measurement with a 2-sided alpha level of 0.05 could be ensured by this sample size using Fisher exact test. Simca 16.1 (Umetrics Inc., Sweden) was used for PCA and partial least square analysis in the groups (control group, HUA group, AGA group, GIP group, and CGA group). SPSS 23.0 (SPSS Inc., Chicago, IL) was used to perform repetitive single-factor ANOVA for the serum samples from the different groups of patients. Later, Pearson correlation coefficients were determined for indexes that may affect the progression of GA by using R language packages (corrplot). By applying the code written by our research team and combining 7 R language packages (quality plan, pROC package, ggplot2 package, mlogit package, RMS package, corrplot package, and survival package), we carried out multiple logistic regression analysis, established a GA diagnosis model, and conducted verification and visualization.

**Table 1:** Baseline characteristics (demographic, living habit, medication, blood biochemical values) of the HUA, AGA, GIP, CGA, RA, and control groups in training set (n = 379).

		GA (n = 267)				
Parameter	Control (n = 80)	HUA (n = 62)	AGA (n = 69)	GIP (n = 74)	CGA (n = 62)	RA (n = 32)
Age, y	52.1 ± 9.3	46.5 ± 15.8	49.6 ± 17.6	44.2 ± 16.2	69.0 ± 6.8^∗∗^^,^^#^	56.3 ± 10.9
BMI, kg/m^2^	22.5 ± 2.6	26.1 ± 1.0^∗∗^^,^^##^	28.9 ± 1.9^∗∗^^,^^##^	25.1 ± 1.4^∗∗^^,^^##^	21.3 ± 1.0^##^	21.7 ± 1.8
UA, μmol/L	147.0 ± 89.4^##^	481.9 ± 60.8^∗∗^^,^^##^	473.0 ± 120.7^∗∗^^,^^##^	420.0 ± 104.8^∗∗^^,^^##^	338.7 ± 76.6^∗∗^^,^^##^	284.3 ± 93.4^∗∗^
BUN, mmol/L	5.0 ± 1.0	5.0 ± 1.4	5.8 ± 3.7	5.1 ± 1.7	8.7 ± 1.9^∗∗^^,^^##^	5.8 ± 2.3
Creatinine, μmol/L	75.7 ± 15.0^#^	82.0 ± 16.3^∗^^,^^#^	101.9 ± 16.7^∗^	104.0 ± 16.1^∗^	211.3 ± 31.0^∗∗^^,^^##^	104.2 ± 21.8^∗^
Hemoglobin, g/L	134.8 ± 8.8	132.8 ± 7.0	135.6 ± 8.5	133.8 ± 7.8	133.2 ± 7.7	135.4 ± 8.5
ESR, mm/h	14.8 ± 3.0^##^	18.1 ± 1.4^#^	30.9 ± 8.0^∗∗^^,^^##^	16.7 ± 1.9^#^	24.1 ± 4.6^∗^	24.9 ± 5.3^∗∗^
HDL, mmol/L	1.5 ± 0.6	1.6 ± 0.5	1.1 ± 0.2^∗∗^^,^^##^	1.0 ± 0.2^∗∗^^,^^##^	1.3 ± 0.3	1.5 ± 0.4
LDL, mmol/L	2.8 ± 0.7	2.5 ± 0.6	2.9 ± 0.9	3.0 ± 0.6	3.0 ± 0.9	2.8 ± 0.8
WBC, 10^9^/L	6.0 ± 1.5^##^	5.7 ± 0.9^##^	8.4 ± 2.8^∗∗^	7.3 ± 1.8^∗^	7.7 ± 1.2^∗∗^	7.5 ± 2.6^∗∗^
TC, mmol/L	5.2 ± 0.9	4.9 ± 1.0	4.9 ± 0.9	5.2 ± 1.2^#^	5.4 ± 0.8	4.7 ± 1.1
TG, mmol/L	1.3 ± 0.7	2.6 ± 1.2^∗∗^^,^^##^	1.9 ± 0.9^∗∗^^,^^##^	2.4 ± 1.5^∗∗^^,^^##^	2.1 ± 1.1^∗∗^^,^^##^	1.2 ± 0.4
CRP, mg/L	0.7 ± 0.8^##^	0.9 ± 0.4^##^	30.5 ± 26.9^∗∗^	6.8 ± 17.9^∗∗^	11.4 ± 6.9^∗∗^^,^^#^	21.9 ± 22.4^∗∗^
RF	<20^##^	<20^##^	<20^##^	<20^##^	<20^##^	290.1 ± 302.1^∗∗^
Smoker (%)	34.4	37.5	55.1^∗∗^^,^^##^	57.3^∗∗^^,^^##^	73.3^∗∗^^,^^##^	20.8
Alcohol drinking habit (%)	25.0^##^	31.9^##^	40.6^∗∗^^,^^##^	42.7^∗∗^^,^^##^	70.0^∗∗^^,^^##^	4.2
Sex (female/male)	35/45	16/46	5/64	4/70	2/60	26/6
Disease duration (months)	/	5.5 ± 3.5	2.0 ± 1.5	1.5 ± 1.0	>132 (11 y)	>60 (5 y)
ULT (febuxostat/benzbromarone/non)	(0/0/160)	(9/1/22)	(42/5/22)	(0/0/74)	(32/0/0)	(0/0/32)
Major disease (tumor/CVD/non)	(0/0/160)	(1/3/28)	(0/0/69)	(0/0/74)	(3/24/3)	(1/2/29)

## 3 Results

### 3.1 Statistical analysis of patient information and serum biochemical indicators

Data, including demographic data, living habits, comorbidities, disease durations, and medical situations, were collected through questionnaires and case histories. The UA level, WBC count, CRP level, BUN level, creatinine level, hemoglobin level, ESR, HDL/LDL level, TC level, and TG level of the participators in each group were determined by the automatic biochemistry analyzer (see Table S1 and Table S2, Supplemental Content, which respectively contain the detailed biochemical index data of patients in the training set and the validation set). The outcome of statistical analysis is provided in Table 1 (training set), and the trends of biochemical indicators in the 5 stages are indicated by box plots (Fig. 1). The RA group (n = 32) was added in this study in order to find a method that could be used to distinguish CGA from RA.

### 3.2 Principal component analysis and orthogonal partial least squares discrimination analysis

**Figure 2:**
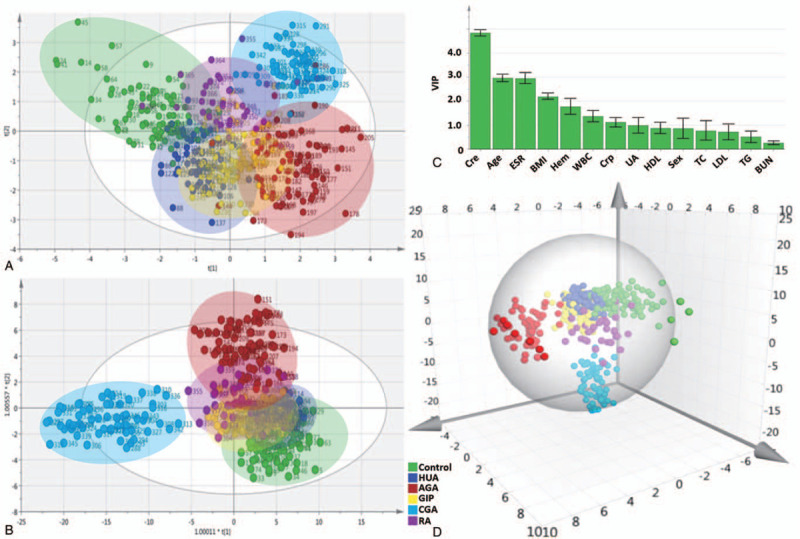
PCA and OPLS-DA results of the serum biochemical profiles of the 6 groups. A represents the PCA score plots, B represents the OPLS-DA score plots, C is the variable important in projection (VIP) value of each biochemical index in the OPLS-DA model, D represents the 3D OPLS-DA score plots. The confidence interval is 95%. The creatinine level, age, ESR, BMI, hemoglobin level, WBC count, CRP level, and UA level contributed significantly to the difference between groups (VIP >1). BMI = body mass index, CRP = C reactive protein, ESR = erythrocyte sedimentation rate; GA = gouty arthritis, OPLS-DA = orthogonal partial least squares discrimination analysis, PCA = principal component analysis, UA = uric acid, VIP = variable important in projection, WBC = white blood cell.

Multivariate analysis was performed on SIMCA-P 16.1 with the raw data of training set (n = 379). In order to determine the intrinsic differences among the 6 groups (control, HUA, AGA, GIP, CGA, and RA), PCA, and OPLS-DA were utilized. The PCA score plots (Fig. 2A) showed a satisfactory separating effect of data among the 6 groups, while R^2^X was 1.00 and *Q*^2^ was 0.978. Moreover, OPLS-DA score plots (Fig. 2B) showed a better consequence, while R^2^X was 0.707, R^2^Y was 0.483, and *Q*^2^ was 0.453. As can be seen from Fig. 2, the biochemical index profile of each group was significantly different from that of the control group, with the greatest difference between the CGA group and the control group. Furthermore, the serum biochemical profiles of CGA and RA were relatively different, indicating that the biochemical indicators selected in this study could adequately distinguish CGA from RA.

### 3.3 Correlation analysis of factors influencing the course of GA

**Figure 3:**
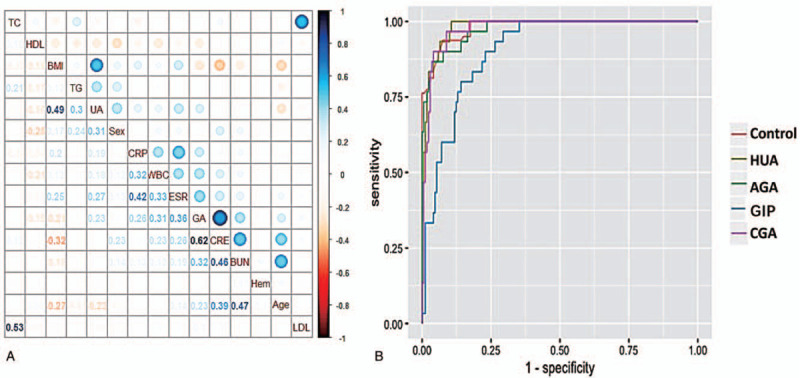
The matrix heat map of the correlation of biochemical indicators (A) and ROC curves (B) associated with the progression of GA. A: Each dot represents a correlation between 2 indicators. The dot size is proportional to the *P* value, and the color gradation of the dots represents the magnitude of the correlation. The number at the bottom left represents the corresponding correlation coefficient with the dot. Blue indicates positive correlation, and red indicates negative correlation. As can be seen, the creatinine level (0.62), ESR (0.36), BUN level (0.32), WBC count (0.31), and UA level (0.23) had the strongest correlations with the progression of GA; B: The areas under the curve (AUCs) of the 5 models are 0.9814 (Control), 0.9288 (HUA), 0.9752 (AGA), 0.9056 (GIP), and 0.9759 (CGA). AGA = acute gouty arthritis, BUN = blood urea nitrogen, CGA = chronic gouty arthritis, ESR = erythrocyte sedimentation rate, GA = gouty arthritis, GIP = during the interictal period, ROC = receiver operating characteristic, UA = uric acid, WBC = white blood cell.

The corrplot package written by our research group in R language was applied for correlation analysis of biochemical indicators associating with progression of GA. The matrix heat map of correlation is shown in Fig. 3A (see Table S3, Supplemental Content, which contains Pearson correlation and Significance). Here, we assume that the GA process is gradually evolved from the control group, HUA group, AGA group, GIP group, and CGA group from light to heavy. As can be seen from Fig. 3A, LDL and TC levels were significantly correlated, indicating that the correlation was not affected by the disease and had little significance for the evolution of GA. In addition, the UA level, creatinine level, WBC count, CRP level, BUN level, ESR, BMI, and HDL level were significantly correlated with the progression of GA, among which the UA level, creatinine level, WBC count, CRP level, BUN level, and ESR were remarkably positively correlated with the progression of GA, while the HDL level and BMI were remarkably negatively correlated with it.

### 3.4 Clinical diagnostic model associating with progression of GA based on serum biochemical indicators

The MASS, PROC, ggplot2, mlogit, RMS, and survival packages in R language were used to carry out ordinal multiple logistic regression analyses for a clinical diagnostic model associated with the progression of GA. The independent variables were without influence upon the regression coefficient and segmentation point of the dependent variables, and there was no multicollinearity between the independent variables. The dependent variable was transformed into the corresponding dummy variable before modeling, and then, the continuous variables (among the independent variables, “sex” was the classified variable and the rest were continuous variables) were standardized, followed by factor analysis. The training set data (n = 379) were adopted to build the model. The 5 stages of GA (control, HUA, AGA, GIP, and CGA) were regarded as dependent variables, and logistic stepwise regression was performed for each stage. Meanwhile, combining the results of “3.1” to “3.4,” risk factors were screened to form the best logistic regression equation for each stage. The prediction probability of the sample under each regression equation was calculated, and the stage of GA in which the maximum probability was located was considered as the diagnosis. The verification set data (n = 200) were adopted to evaluate and verify the model. Details of the clinical diagnostic model of GA are provided in Table 2.

**Table 2:** Details of clinical diagnostic model of GA.

Model	Factors	OR	95% CI	*P* value	Regression equation
Model Control	UA	0.97	0.96–0.98	2.21 × 10^−15^	$P_{1}=1/1+{\text{e}}^{-Z1}$
	CRP	8.28	3.91–19.34	1.93 × 10^–7^	
Model HUA	HDL	1.01	1.01–1.02	1.24 × 10^−12^	$P_{2}=1/1+{\text{e}}^{-Z2}$
	UA	0.95	0.92–0.97	6.29 × 10^−7^	
	WBC	1.05	1.03–1.07	1.85 × 10^–6^	
Model AGA	UA	1.01	1.01–1.02	4.17 × 10^−8^	$P_{3}=1/1+{\text{e}}^{-Z3}$
	ESR	1.33	1.24–1.45	3.20 × 10^−13^	
	WBC	1.20	1.01–1.43	3.78 × 10^–2^	
Model GIP	HDL	0.03	0.007–0.09	5.33 × 10^−8^	$P_{4}=1/1+{\text{e}}^{-Z4}$
	UA	1.01	1.003–1.009	1.15 × 10^−6^	
	ESR	0.73	0.66–0.80	2.76 × 10^−9^	
Model CGA	BUN	1.83	1.58–2.15	2.52 × 10^−14^	$P_{5}=1/1+{\text{e}}^{-Z5}$
	ESR	1.04	1.00–1.09	5.29 × 10^−2^	

### 3.5 Evaluation and verification of a clinical diagnostic model associated with the progression of GA

The validation set data were substituted into the above models, and the predicted results were outputted and compared with the actual results. The receiver operating characteristic (ROC) curves of 5 models are shown in Fig. 3B. The total area under the curve (AUC) of the clinical diagnostic model associated with the progression of GA was 0.9534, and the kappa coefficient was applied to evaluate the consistency between the predicted results of the model and the actual results. Encouragingly, the kappa coefficient of the clinical diagnostic model was 0.80, which indicated a substantial consistency of this model (the magnitude of the kappa coefficient could be divided into 5 degrees as follows: 0.0–0.20, slight consistency; 0.21–0.40, fair consistency; 0.41–0.60, moderate consistency; 0.61–0.80, substantial consistency; 0.81–1, almost perfect consistency). It shows that the model has high accuracy and reliability.

### 3.6 Visualizing the clinical diagnostic model associated with the progression of GA

In order to achieve greater application of the GA diagnostic model in clinical practice, a nomogram of the clinical diagnostic model associated with the progression of GA was made by adopting the R language rms package (see Figure S3, Supplemental Content, which demonstrates the nomogram of 5 models. A: Control group, B: HUA group, C: AGA group, D: GIP group, E: CGA group). According to the regression coefficient of each influencing factor in the multiple regression model, each influencing factor was scored. The summation function of the score was converted into the probability of the occurrence of the outcome event, and the stage of the maximum probability was the diagnosis result. This method can transform the complex regression equation into a simple and visual chart, making the results of the prediction model more readable and useful.

## 4 Discussion

Statistical studies showed that the prevalence of GA is increasing every year, especially in developing countries, with a 2% increase in the prevalence of GA from 2017 to 2019.^[23,24]^ The incidence of GA in European and American countries is 0.13% to 0.37%, and the annual incidence is 0.20% to 0.35%.^[25]^ The prevalence of HUA in the general population in China is about 10%.^[26]^ So far, >80% of the published articles on disease prediction models for arthritis were associated with RA,^[27–30]^ according to our literature review and document retrieval. Thus, it could be seen that GA is not a research hotspot in the field of arthritis, which is not commensurate with its high incidence. In order to reverse this abnormal phenomenon, the present study assessed a clinical diagnostic model associated with the progression of GA based on serum biochemical indicators.

We found a strong correlation between LDL and TC levels in all groups (LDL/TC in the control, HUA, AGA, GIP, and CGA groups: 0.54, 0.51, 0.59, 0.58, and 0.55), which is consistent with literature reports.^[31]^ The correlation was not affected by GA progression, which indicated that LDL and TC were not independent risk factors (IRFs) of the progression of GA. Moreover, according to line charts in Fig. 1, hemoglobin tended to flatten out in the 5 stages of GA. Therefore, LDL, TC, and hemoglobin levels were excluded in the establishment of a diagnostic model.

Box plots C and I in Fig. 1 clearly indicated that BUN and creatinine levels showed significant elevation in the CGA group compared with the other 5 groups. The kidney is the main organ for excreting BUN, and as with serum creatinine, BUN could be in the normal range in the early stages of renal function impairment. However, BUN and creatinine levels would rise rapidly when the glomerular filtration rate (GFR) drops below 50% of the normal rate. Studies have shown that chronically high UA levels in the blood could significantly increase the risk of kidney disease,^[32]^ and 5.6% of 13,338 participants (mean serum UA level = 5.9 ± 1.5 mg/dL) had incident kidney disease defined by a GFR decrease of >30% over 8.5 years.^[33]^ Another population-based cohort study showed that patients with CGA who were treated with ULT had a greater risk of incident chronic kidney disease.^[34]^ Therefore, BUN and creatinine could be regarded as IRFs for the progression of GA, and significant increases in BUN and creatinine levels indicate that the course of GA has entered the CGA stage. However, there were no significant differences in BUN and creatinine levels among the control, HUA, AGA, and GIP groups, so the establishment of a clinical diagnostic model for the progression of GA requires the contribution of other indicators. Besides, BUN and creatinine could be considered as diagnostic indicators of CGA and are serum biochemical indicators that could distinguish CGA from RA in addition to RF.

Moreover, as can be seen in Fig. 2, the biochemical profile of each group was significantly different from that of the control group, with the greatest difference between the CGA group and the control group, and the creatinine level, age, ESR, BMI, hemoglobin level, WBC count, CRP level, and UA level contributed significantly to the difference between groups (VIP >1). Furthermore, the serum biochemical profiles of CGA and RA were relatively different, indicating that the biochemical indicators selected in this study could adequately distinguish the 6 groups including the 5 GA stages and RA. On this account, we suppose that the bias of the overall profile of the patient's serum biochemical indicators to a certain stage may lead to the appearance of symptoms. Therefore, we believe that the previous single indicator diagnosis method should be improved, and the approach should adopt multiple indicators for comprehensive clinical diagnosis and prediction. Meanwhile, according to the results of correlation analysis, the UA level, creatinine level, WBC count, CRP level, BUN level, TG level, ESR, and HDL level were significantly correlated with the progression of GA, among which the creatinine level (0.62), ESR (0.36), BUN level (0.32), WBC count (0.31), and UA level (0.23) had the strongest correlations with it. The findings are consistent with the results of multivariate statistics.

The training set data (n = 379) were adopted to build the clinical diagnostic model associated with the progression of GA (consists of 5 phases), and the verification set data (n = 200) were adopted to evaluate and verify it. The AUCs of the 5 models were 0.9814 (Control), 0.9288 (HUA), 0.9752 (AGA), 0.9056 (GIP), and 0.9759 (CGA). The kappa coefficient applied to evaluate the consistency was 0.80, indicating substantial consistency of this model. Furthermore, the visualization of the model is realized by using nomograms, which facilitates more clinical application of the model. Both doctors and patients can judge the development and changes of GA according to the model, which provides an effective reference for distinguishing the development stages of GA.

After this study, non-targeted metabolomics analysis will be performed on the serum samples of GA patients collected above to obtain potential diagnostic biomarkers related to the progression of GA, including absolutely qualitative and quantitative verification. The changes in biomarkers and serum biochemical indexes in different progressions of GA were found to optimize the clinical diagnostic model of GA based on the serum biochemical indexes proposed in this paper. There is an expectation to develop a new clinical diagnosis method for GA similar to the “GA diagnostic kit” which can help clinicians predict the progress of GA faster and more accurately.

## 5 Conclusion

In this study, serum biochemical indicators from clinical assessment were adopted to establish a clinical diagnostic model and serum biochemical profile associated with the progression of GA. It is the first study to investigate the serum biochemical profile at different stages of GA. An obvious difference in the serum biochemical profile among GA stages was found in this paper, which could effectively distinguish them. The hypothetical algorithm of GA diagnostic model was *P* = 1/1 + *e*^–*Z*^, in which *P* stands for prediction probability, *Z* stands for independent risk factor variables at each stage of GA (health: 5.38–0.0251 × UA; HUA: 4.12 + 2.11 × HDL + 0.0114 × UA − 0.0560 × CRP; AGA: 14.8 + 0.0464 × CRP + 0.0132 × UA + 0.287 × ESR; GIP: 4.92 + 0.184 × WBC − 3.52 × HDL + 0.00608 × UA − 0.310 × ESR; CGA: 6.58 + 0.602 × BUN + 0.0436 × ESR). The prediction probability of the sample under each regression equation was calculated, and the stage of GA in which the maximum probability was located was considered as the diagnosis. We suppose that the bias of the overall profile of a patient's serum biochemical indicators to a certain stage may lead to the appearance of symptoms. A simple evaluation tool was developed, and we have reason to believe it will be useful for clinicians and researchers wishing to consolidate their clinical diagnosis in regard to the stage and tendency of GA patients. This clinical diagnostic model could be applied clinically and in research to improve the accuracy of the identification and prediction of these stages of GA patients.

## Author contributions

**Conceptualization:** Shang Lyu, Shilin Yang, Peng Liu.

**Data curation:** Ruowen Ding, Hui Ouyang.

**Funding acquisition:** Shilin Yang, Yulin Feng.

**Investigation:** Shang Lyu, Ruowen Ding, Wanyuan Chen, Peng Liu.

**Software:** Yi Rao.

**Validation:** Shilin Yang.

**Visualization:** Ruowen Ding.

**Writing – original draft:** Shang Lyu.

## Supplementary Material

Supplemental Digital Content

## Supplementary Material

Supplemental Digital Content

## Supplementary Material

Supplemental Digital Content

## Supplementary Material

Supplemental Digital Content

## Supplementary Material

Supplemental Digital Content

## Supplementary Material

Supplemental Digital Content
