# Supplementary figures and images for: Establishment of a clinical diagnostic model for gouty arthritis based on the serum biochemical profile: A case-control study
Source: Medicine (Baltimore). 2021 Apr 23;100(16):e25542. doi: 10.1097/MD.0000000000025542 (PMC8078334; doi:10.1097/MD.0000000000025542)

Figure S1: Abridged general view of the evolution of gouty arthritis.


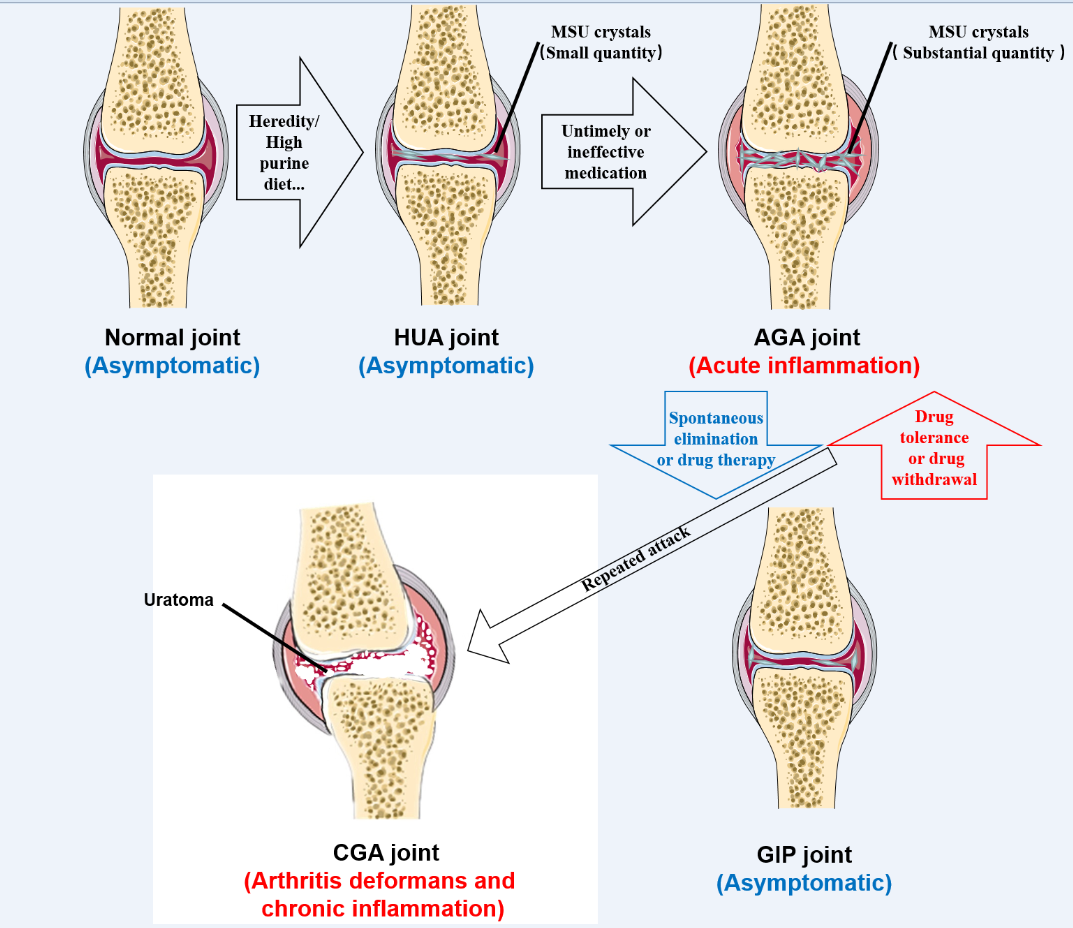

Supplement: Supplemental Digital Content [file medi-100-e25542-s001.doc]

Figure S2: The overview of study design.


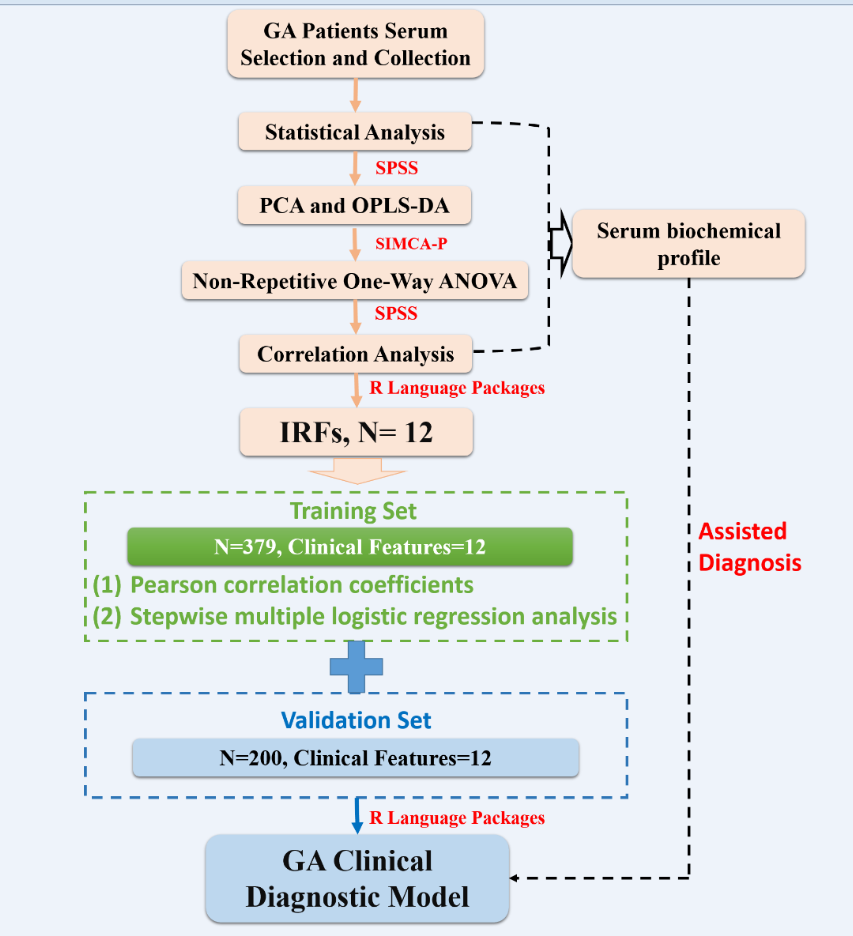

Supplement: Supplemental Digital Content [file medi-100-e25542-s002.doc]

Figure S3: The nomogram of five models. A: Control group, B: HUA group, C: AGA group, D: DIP group, E: CGA group.


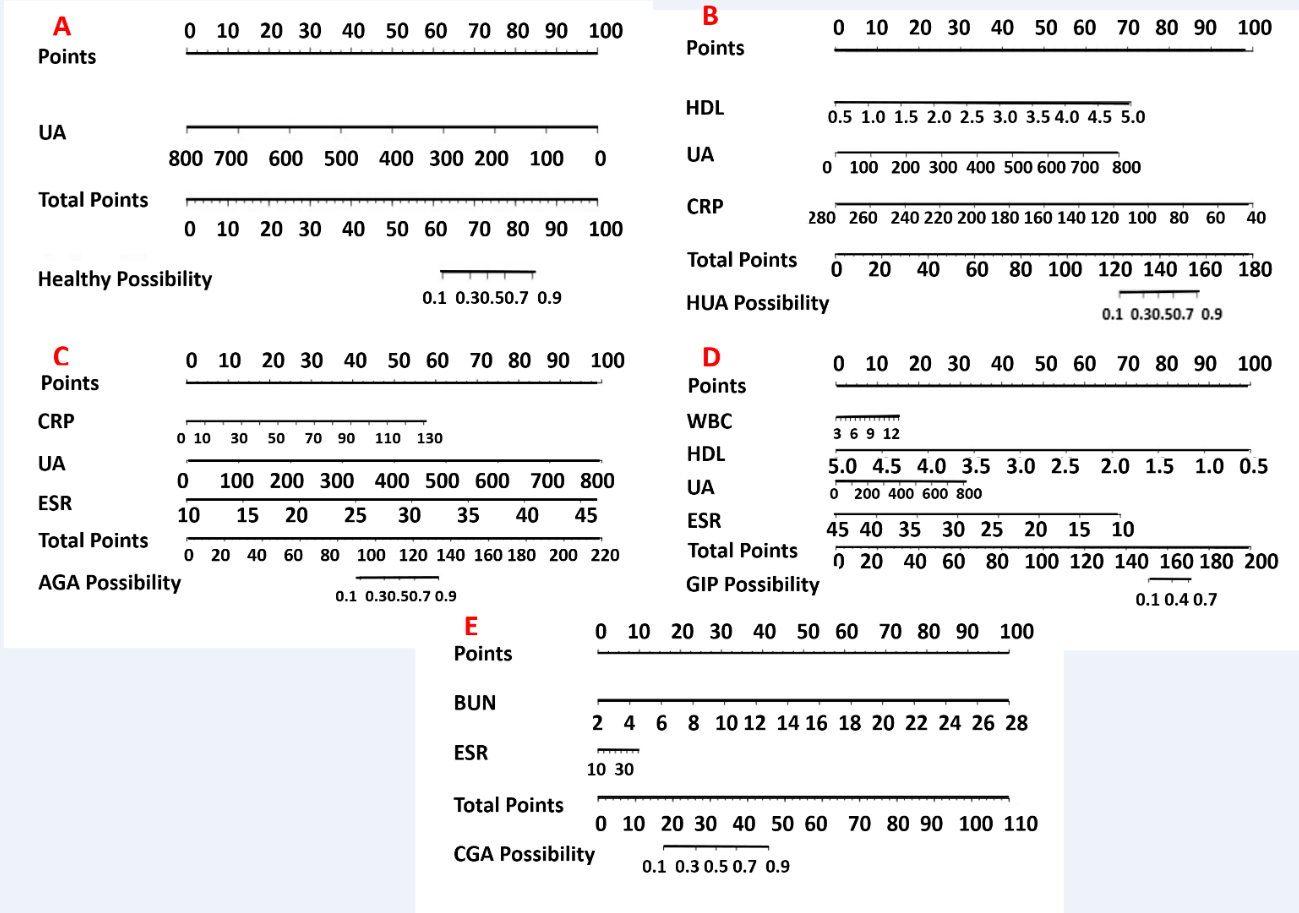

Supplement: Supplemental Digital Content [file medi-100-e25542-s006.doc]
