# Supplementary material for: Establishment of a clinical diagnostic model for gouty arthritis based on the serum biochemical profile: A case-control study
Source: Medicine (Baltimore). 2021 Apr 23;100(16):e25542. doi: 10.1097/MD.0000000000025542 (PMC8078334; doi:10.1097/MD.0000000000025542)
Supplement: Supplemental Digital Content [file medi-100-e25542-s003.doc]

**Table S3 . Detailed correlation analysis data**

|  | | WBC | CRP | BUN | HDL | LDL | TC | TG | BMI | UA | Sex | Age | Cre | Hem | ESR | GA |
| --- | --- | --- | --- | --- | --- | --- | --- | --- | --- | --- | --- | --- | --- | --- | --- | --- |
| WBC | Pearson correlation | 1 | .325^**^ | .131^*^ | -.210^**^ | .072 | -.035 | .062 | .124^*^ | .176^**^ | .121^*^ | .007 | .228^**^ | -.005 | .332^**^ | .313^**^ |
|  | Significance(2-sided) |  | .000 | .011 | .000 | .161 | .495 | .230 | .016 | .001 | .019 | .897 | .000 | .926 | .000 | .000 |
| CRP | Pearson correlation | .325^**^ | 1 | .125^*^ | -.137^**^ | -.052 | -.136^**^ | -.063 | .201^**^ | .192^**^ | .029 | .040 | .089 | .037 | .423^**^ | .264^**^ |
|  | Significance(2-sided) | .000 |  | .015 | .007 | .313 | .008 | .222 | .000 | .000 | .579 | .438 | .085 | .471 | .000 | .000 |
| BUN | Pearson correlation | .131^*^ | .125^*^ | 1 | -.027 | -.045 | -.047 | -.060 | -.181^**^ | -.007 | .143^**^ | .469^**^ | .464^**^ | .109^*^ | .187^**^ | .320^**^ |
|  | Significance(2-sided) | .011 | .015 |  | .604 | .380 | .362 | .244 | .000 | .894 | .005 | .000 | .000 | .035 | .000 | .000 |
| HDL | Pearson correlation | -.210^**^ | -.137^**^ | -.027 | 1 | -.105^*^ | .117^*^ | -.172^**^ | -.188^**^ | -.192^**^ | -.249^**^ | .094 | -.064 | -.043 | -.122^*^ | -.190^**^ |
|  | Significance(2-sided) | .000 | .007 | .604 |  | .041 | .022 | .001 | .000 | .000 | .000 | .068 | .212 | .399 | .017 | .000 |
| LDL | Pearson correlation | .072 | -.052 | -.045 | -.105^*^ | 1 | .531^**^ | .006 | -.068 | -.083 | -.077 | -.002 | .086 | -.070 | .070 | .103^*^ |
|  | Significance(2-sided) | .161 | .313 | .380 | .041 |  | .000 | .908 | .189 | .107 | .132 | .965 | .095 | .176 | .171 | .045 |
| TC | Pearson correlation | -.035 | -.136^**^ | -.047 | .117^*^ | .531^**^ | 1 | .215^**^ | -.132^*^ | -.099 | -.049 | .033 | .119^*^ | -.069 | -.028 | .017 |
|  | Significance(2-sided) | .495 | .008 | .362 | .022 | .000 |  | .000 | .010 | .053 | .339 | .526 | .021 | .183 | .589 | .736 |
| TG | Pearson correlation | .062 | -.063 | -.060 | -.172^**^ | .006 | .215^**^ | 1 | .121^*^ | .305^**^ | .239^**^ | -.148^**^ | .046 | -.045 | -.013 | .049 |
|  | Significance(2-sided) | .230 | .222 | .244 | .001 | .908 | .000 |  | .018 | .000 | .000 | .004 | .377 | .386 | .806 | .344 |
| BMI | Pearson correlation | .124^*^ | .201^**^ | -.181^**^ | -.188^**^ | -.068 | -.132^*^ | .121^*^ | 1 | .492^**^ | .168^**^ | -.273^**^ | -.318^**^ | .027 | .253^**^ | -.211^**^ |
|  | Significance(2-sided) | .016 | .000 | .000 | .000 | .189 | .010 | .018 |  | .000 | .001 | .000 | .000 | .597 | .000 | .000 |
| UA | Pearson correlation | .176^**^ | .192^**^ | -.007 | -.192^**^ | -.083 | -.099 | .305^**^ | .492^**^ | 1 | .306^**^ | -.223^**^ | .043 | -.052 | .274^**^ | .232^**^ |
|  | Significance(2-sided) | .001 | .000 | .894 | .000 | .107 | .053 | .000 | .000 |  | .000 | .000 | .402 | .313 | .000 | .000 |
| Sex | Pearson correlation | .121^*^ | .029 | .143^**^ | -.249^**^ | -.077 | -.049 | .239^**^ | .168^**^ | .306^**^ | 1 | -.083 | .228^**^ | -.067 | .127^*^ | .065 |
|  | Significance(2-sided) | .019 | .579 | .005 | .000 | .132 | .339 | .000 | .001 | .000 |  | .108 | .000 | .192 | .014 | .207 |
| Age | Pearson correlation | .007 | .040 | .469^**^ | .094 | -.002 | .033 | -.148^**^ | -.273^**^ | -.223^**^ | -.083 | 1 | .391^**^ | .098 | .143^**^ | .235^**^ |
|  | Significance(2-sided) | .897 | .438 | .000 | .068 | .965 | .526 | .004 | .000 | .000 | .108 |  | .000 | .057 | .005 | .000 |
| Cre | Pearson correlation | .228^**^ | .089 | .464^**^ | -.064 | .086 | .119^*^ | .046 | -.318^**^ | .043 | .228^**^ | .391^**^ | 1 | -.042 | .260^**^ | .618^**^ |
|  | Significance(2-sided) | .000 | .085 | .000 | .212 | .095 | .021 | .377 | .000 | .402 | .000 | .000 |  | .412 | .000 | .000 |
| Hem | Pearson correlation | -.005 | .037 | .109^*^ | -.043 | -.070 | -.069 | -.045 | .027 | -.052 | -.067 | .098 | -.042 | 1 | .056 | -.011 |
|  | Significance(2-sided) | .926 | .471 | .035 | .399 | .176 | .183 | .386 | .597 | .313 | .192 | .057 | .412 |  | .280 | .838 |
| ESR | Pearson correlation | .332^**^ | .423^**^ | .187^**^ | -.122^*^ | .070 | -.028 | -.013 | .253^**^ | .274^**^ | .127^*^ | .143^**^ | .260^**^ | .056 | 1 | .363^**^ |
|  | Significance(2-sided) | .000 | .000 | .000 | .017 | .171 | .589 | .806 | .000 | .000 | .014 | .005 | .000 | .280 |  | .000 |
| GA | Pearson correlation | .313^**^ | .264^**^ | .320^**^ | -.190^**^ | .103^*^ | .017 | .049 | -.211^**^ | .232^**^ | .065 | .235^**^ | .618^**^ | -.011 | .363^**^ | 1 |
|  | Significance(2-sided) | .000 | .000 | .000 | .000 | .045 | .736 | .344 | .000 | .000 | .207 | .000 | .000 | .838 | .000 |  |

* means *p* < 0.05, ** means *p* < 0.01.
