# Supplementary material for: Establishment of a clinical diagnostic model for gouty arthritis based on the serum biochemical profile: A case-control study
Source: Medicine (Baltimore). 2021 Apr 23;100(16):e25542. doi: 10.1097/MD.0000000000025542 (PMC8078334; doi:10.1097/MD.0000000000025542)
Supplement: Supplemental Digital Content [file medi-100-e25542-s004.doc]

**Table S1. Raw data of biochemical indicators in serum specimens of training set（n=379）**

| **NO.** | **WBC**  **(10∧9/L)** | **CRP**  **(Mg/L)** | **BUN**  **(mmol/L)** | **HDL**  **(mmol/L)** | **LDL**  **(mmol/L)** | **TC**  **(mmol/L)** | **TG**  **(mmol/L)** | **RF** | **BMI**  **(kg/m^2^)** | **UA**  **(μmol/L)** | **Sex** | **Age** | **Creatinine**  **(μmol/L)** | **Hemoglobin**  **(g/L)** | **ESR**  **(mm/h)** |
| --- | --- | --- | --- | --- | --- | --- | --- | --- | --- | --- | --- | --- | --- | --- | --- |
| **Control group** | | | | | | | | | | | | | | | |
| Control-001 | 4.60 | 0.30 | 5.35 | 1.68 | 3.13 | 5.79 | 0.83 | <20 | 22.66 | 108 | female | 55 | 77.16 | 141.60 | 18.75 |
| Control-002 | 6.41 | 0.30 | 6.58 | 1.85 | 2.05 | 4.64 | 0.58 | <20 | 25.24 | 107 | female | 48 | 88.16 | 125.49 | 12.89 |
| Control-003 | 10.12 | 3.65 | 7.01 | 1.90 | 3.40 | 6.22 | 1.07 | <20 | 23.12 | 149 | female | 56 | 72.88 | 135.30 | 11.02 |
| Control-004 | 6.35 | 0.30 | 5.35 | 0.91 | 1.82 | 3.42 | 1.48 | <20 | 19.15 | 104 | male | 44 | 71.63 | 126.06 | 18.58 |
| Control-005 | 6.70 | 0.30 | 3.17 | 2.09 | 1.68 | 4.46 | 0.86 | <20 | 18.80 | 147 | female | 42 | 52.30 | 134.10 | 12.66 |
| Control-006 | 7.45 | 0.30 | 4.51 | 1.35 | 3.17 | 5.36 | 1.98 | <20 | 22.80 | 145 | male | 42 | 90.89 | 144.75 | 15.63 |
| Control-007 | 5.68 | 0.30 | 5.26 | 1.82 | 2.59 | 5.47 | 0.76 | <20 | 18.96 | 97 | female | 43 | 73.83 | 122.79 | 14.69 |
| Control-008 | 6.12 | 0.30 | 4.86 | 1.57 | 2.62 | 4.96 | 0.83 | <20 | 25.10 | 136 | male | 44 | 73.62 | 133.83 | 14.99 |
| Control-009 | 5.13 | 0.30 | 7.83 | 1.58 | 3.13 | 5.29 | 0.66 | <20 | 18.61 | 122 | male | 63 | 69.44 | 145.78 | 10.79 |
| Control-010 | 5.80 | 0.30 | 5.47 | 0.98 | 2.13 | 4.01 | 1.63 | <20 | 23.23 | 182 | male | 58 | 79.13 | 135.00 | 13.61 |
| Control-011 | 7.46 | 1.47 | 4.84 | 1.92 | 2.87 | 5.93 | 1.70 | <20 | 19.96 | 96 | male | 56 | 98.44 | 123.86 | 10.73 |
| Control-012 | 8.36 | 1.80 | 4.58 | 1.00 | 3.44 | 5.69 | 2.38 | <20 | 21.11 | 152 | male | 48 | 92.45 | 127.14 | 13.71 |
| Control-013 | 3.86 | 0.30 | 5.14 | 1.12 | 3.07 | 5.23 | 1.47 | <20 | 25.24 | 101 | male | 45 | 94.71 | 123.62 | 10.86 |
| Control-014 | 5.11 | 0.30 | 4.97 | 4.56 | 2.17 | 4.48 | 0.75 | <20 | 18.89 | 113 | male | 43 | 91.09 | 135.62 | 14.67 |
| Control-015 | 8.36 | 2.09 | 4.58 | 1.00 | 3.44 | 5.69 | 2.38 | <20 | 19.60 | 126 | male | 59 | 79.52 | 146.49 | 16.04 |
| Control-016 | 6.01 | 0.30 | 5.01 | 1.10 | 1.77 | 3.52 | 0.75 | <20 | 22.10 | 125 | male | 67 | 54.79 | 149.03 | 18.93 |
| Control-017 | 6.90 | 0.30 | 5.74 | 1.52 | 3.68 | 6.01 | 1.12 | <20 | 20.67 | 167 | female | 51 | 87.73 | 132.12 | 16.14 |
| Control-018 | 6.31 | 0.30 | 3.98 | 1.44 | 2.88 | 5.01 | 1.20 | <20 | 19.87 | 168 | female | 46 | 66.50 | 145.95 | 13.09 |
| Control-019 | 4.02 | 0.30 | 6.14 | 1.22 | 3.23 | 5.89 | 1.94 | <20 | 21.02 | 128 | male | 49 | 59.22 | 137.21 | 16.05 |
| Control-020 | 5.60 | 0.30 | 5.10 | 1.29 | 2.81 | 4.98 | 1.06 | <20 | 22.62 | 132 | male | 42 | 53.54 | 122.40 | 14.61 |
| Control-021 | 6.59 | 0.30 | 5.23 | 1.46 | 3.50 | 5.60 | 1.35 | <20 | 19.69 | 193 | female | 57 | 80.60 | 132.29 | 16.48 |
| Control-022 | 3.54 | 0.30 | 5.74 | 2.29 | 2.11 | 5.14 | 0.49 | <20 | 25.80 | 130 | male | 76 | 69.07 | 140.44 | 17.81 |
| Control-023 | 9.98 | 2.90 | 5.39 | 1.50 | 4.15 | 7.15 | 2.80 | <20 | 21.92 | 217 | male | 60 | 61.31 | 146.77 | 12.93 |
| Control-024 | 5.14 | 0.30 | 4.52 | 2.35 | 2.04 | 5.42 | 0.80 | <20 | 20.93 | 5 | female | 41 | 76.77 | 140.28 | 17.13 |
| Control-025 | 6.61 | 0.30 | 4.93 | 1.00 | 2.83 | 4.86 | 1.11 | <20 | 23.76 | 157 | male | 45 | 53.52 | 125.00 | 15.67 |
| Control-026 | 7.81 | 0.30 | 4.64 | 1.08 | 2.02 | 4.08 | 1.78 | <20 | 24.53 | 39 | male | 61 | 51.07 | 143.15 | 18.98 |
| Control-027 | 8.13 | 1.45 | 5.18 | 1.20 | 2.85 | 5.36 | 2.01 | <20 | 18.52 | 33 | male | 48 | 51.62 | 139.53 | 18.77 |
| Control-028 | 4.93 | 0.30 | 3.60 | 2.13 | 2.58 | 5.62 | 0.80 | <20 | 22.92 | 65 | female | 50 | 98.80 | 137.34 | 19.68 |
| Control-029 | 4.58 | 0.30 | 4.40 | 1.69 | 2.11 | 4.45 | 0.77 | <20 | 27.56 | 68 | female | 52 | 58.08 | 129.85 | 17.86 |
| Control-030 | 4.54 | 0.30 | 4.02 | 1.46 | 3.39 | 5.90 | 1.30 | <20 | 21.61 | 231 | female | 49 | 75.79 | 126.77 | 10.84 |
| Control-031 | 5.23 | 0.30 | 4.28 | 1.51 | 2.39 | 4.92 | 1.34 | <20 | 24.37 | 241 | female | 44 | 60.61 | 130.18 | 12.97 |
| Control-032 | 4.73 | 0.30 | 5.50 | 1.38 | 3.10 | 5.15 | 1.10 | <20 | 20.57 | 302 | male | 56 | 80.90 | 120.43 | 17.27 |
| Control-033 | 6.82 | 0.30 | 5.47 | 1.17 | 2.87 | 5.00 | 1.95 | <20 | 18.77 | 27 | male | 47 | 90.08 | 140.82 | 10.92 |
| Control-034 | 4.18 | 0.30 | 2.72 | 2.22 | 3.57 | 6.66 | 0.78 | <20 | 18.61 | 304 | female | 44 | 80.51 | 148.62 | 10.09 |
| Control-035 | 6.19 | 0.30 | 3.83 | 1.36 | 2.78 | 5.13 | 2.16 | <20 | 22.21 | 107 | male | 42 | 84.10 | 122.95 | 12.01 |
| Control-036 | 6.35 | 0.30 | 5.28 | 1.04 | 2.38 | 4.27 | 1.98 | <20 | 27.60 | 277 | male | 52 | 93.33 | 121.06 | 13.97 |
| Control-037 | 7.79 | 0.30 | 3.53 | 1.41 | 1.81 | 4.24 | 2.02 | <20 | 20.85 | 306 | male | 44 | 62.72 | 123.22 | 14.69 |
| Control-038 | 8.21 | 1.54 | 5.09 | 1.06 | 2.01 | 3.72 | 0.65 | <20 | 26.70 | 59 | male | 55 | 96.68 | 129.42 | 17.75 |
| Control-039 | 6.20 | 0.60 | 7.23 | 0.85 | 2.79 | 5.11 | 3.13 | <20 | 22.71 | 49 | male | 46 | 93.71 | 149.22 | 15.25 |
| Control-040 | 6.00 | 0.60 | 4.46 | 1.48 | 3.72 | 6.06 | 1.11 | <20 | 24.48 | 321 | female | 42 | 61.17 | 128.71 | 17.84 |
| Control-041 | 4.25 | 0.60 | 4.93 | 1.77 | 2.48 | 4.72 | 0.80 | <20 | 21.76 | 3 | female | 42 | 64.11 | 147.78 | 13.79 |
| Control-042 | 4.91 | 0.60 | 4.59 | 0.94 | 2.91 | 4.63 | 1.23 | <20 | 24.44 | 78 | male | 42 | 66.29 | 127.89 | 11.49 |
| Control-043 | 5.05 | 0.60 | 3.60 | 1.82 | 4.01 | 6.83 | 0.80 | <20 | 20.21 | 225 | female | 52 | 94.10 | 129.37 | 11.31 |
| Control-044 | 4.49 | 0.60 | 4.16 | 1.71 | 3.97 | 6.52 | 0.83 | <20 | 26.51 | 231 | male | 68 | 58.93 | 120.94 | 15.69 |
| Control-045 | 5.96 | 0.60 | 6.52 | 3.66 | 4.46 | 5.54 | 1.22 | <20 | 21.60 | 2 | male | 42 | 96.69 | 146.92 | 19.90 |
| Control-046 | 6.21 | 0.60 | 3.93 | 1.24 | 4.03 | 6.72 | 2.84 | <20 | 18.68 | 230 | female | 47 | 53.17 | 125.31 | 11.86 |
| Control-047 | 5.25 | 0.50 | 5.43 | 1.50 | 2.01 | 4.12 | 0.84 | <20 | 23.15 | 276 | male | 70 | 96.72 | 143.26 | 16.27 |
| Control-048 | 4.75 | 0.50 | 5.67 | 1.32 | 2.72 | 4.56 | 0.61 | <20 | 25.12 | 281 | male | 60 | 65.15 | 146.47 | 16.61 |
| Control-049 | 5.54 | 0.50 | 4.47 | 1.51 | 2.37 | 4.44 | 1.02 | <20 | 22.57 | 271 | female | 69 | 83.44 | 140.51 | 13.12 |
| Control-050 | 3.14 | 0.50 | 4.76 | 1.23 | 2.33 | 4.14 | 0.79 | <20 | 24.14 | 298 | male | 50 | 71.31 | 141.36 | 16.31 |
| Control-051 | 5.27 | 0.50 | 3.61 | 1.71 | 2.32 | 4.63 | 0.84 | <20 | 24.22 | 209 | male | 65 | 83.81 | 138.59 | 14.21 |
| Control-052 | 5.76 | 0.50 | 4.54 | 1.32 | 2.97 | 5.24 | 1.23 | <20 | 19.35 | 314 | male | 44 | 53.67 | 140.54 | 19.85 |
| Control-053 | 7.77 | 0.50 | 4.39 | 1.58 | 3.13 | 5.87 | 1.98 | <20 | 22.45 | 308 | male | 42 | 86.16 | 145.70 | 16.98 |
| Control-054 | 5.02 | 0.50 | 7.30 | 1.90 | 2.90 | 5.77 | 0.67 | <20 | 22.26 | 202 | male | 71 | 81.57 | 122.62 | 15.68 |
| Control-055 | 5.34 | 0.50 | 5.30 | 1.43 | 3.02 | 5.22 | 0.62 | <20 | 19.21 | 306 | female | 52 | 89.42 | 131.28 | 19.21 |
| Control-056 | 7.49 | 0.50 | 4.76 | 1.07 | 3.35 | 5.19 | 0.94 | <20 | 27.24 | 33 | female | 53 | 87.49 | 145.09 | 14.73 |
| Control-057 | 5.17 | 0.60 | 6.93 | 3.13 | 2.11 | 6.61 | 1.00 | <20 | 21.01 | 29 | male | 58 | 95.54 | 133.50 | 14.07 |
| Control-058 | 3.41 | 0.60 | 5.80 | 1.77 | 2.65 | 5.27 | 0.70 | <20 | 22.27 | 38 | female | 63 | 79.17 | 127.51 | 10.10 |
| Control-059 | 5.01 | 0.60 | 5.75 | 0.80 | 2.48 | 4.00 | 1.39 | <20 | 23.34 | 339 | male | 69 | 89.05 | 125.52 | 13.92 |
| Control-060 | 8.90 | 2.60 | 3.87 | 0.98 | 2.39 | 4.71 | 4.14 | <20 | 24.12 | 57 | male | 41 | 59.91 | 127.59 | 17.48 |
| Control-061 | 5.21 | 0.60 | 6.92 | 1.15 | 3.00 | 5.02 | 1.30 | <20 | 20.30 | 50 | male | 46 | 69.02 | 144.49 | 11.05 |
| Control-062 | 11.08 | 4.60 | 3.58 | 1.42 | 2.70 | 4.83 | 1.07 | <20 | 27.86 | 23 | female | 46 | 87.00 | 129.79 | 19.27 |
| Control-063 | 5.26 | 0.50 | 5.33 | 1.45 | 1.96 | 4.10 | 0.81 | <20 | 24.59 | 79 | female | 47 | 51.53 | 133.44 | 10.02 |
| Control-064 | 4.63 | 0.30 | 5.00 | 1.70 | 4.00 | 6.64 | 1.20 | <20 | 19.93 | 46 | female | 47 | 50.91 | 146.66 | 18.53 |
| Control-065 | 6.81 | 0.80 | 5.25 | 1.24 | 3.48 | 5.73 | 1.06 | <20 | 22.10 | 90 | female | 49 | 58.94 | 139.48 | 17.84 |
| Control-066 | 5.97 | 0.30 | 5.86 | 1.32 | 3.15 | 6.22 | 3.07 | <20 | 19.00 | 84 | male | 59 | 73.51 | 128.61 | 17.59 |
| Control-067 | 5.01 | 0.30 | 5.52 | 1.94 | 2.69 | 5.61 | 0.69 | <20 | 25.63 | 58 | male | 50 | 74.60 | 122.36 | 19.82 |
| Control-068 | 5.56 | 0.30 | 3.36 | 1.91 | 2.48 | 5.52 | 1.30 | <20 | 25.47 | 113 | female | 48 | 92.46 | 127.54 | 12.36 |
| Control-069 | 4.23 | 0.30 | 3.54 | 0.90 | 2.15 | 4.10 | 1.73 | <20 | 19.84 | 99 | female | 72 | 81.30 | 133.31 | 12.59 |
| Control-070 | 4.84 | 0.80 | 5.69 | 1.08 | 3.15 | 5.01 | 1.18 | <20 | 23.57 | 63 | male | 57 | 85.23 | 142.66 | 12.05 |
| Control-071 | 5.73 | 0.80 | 5.80 | 1.26 | 3.03 | 5.19 | 1.64 | <20 | 24.47 | 133 | female | 81 | 52.69 | 141.79 | 18.73 |
| Control-072 | 5.84 | 0.80 | 5.84 | 1.73 | 2.71 | 5.36 | 0.71 | <20 | 23.63 | 172 | female | 49 | 97.41 | 134.99 | 10.29 |
| Control-073 | 5.69 | 0.80 | 4.38 | 1.24 | 2.99 | 4.91 | 1.15 | <20 | 22.93 | 165 | male | 46 | 93.98 | 147.59 | 10.78 |
| Control-074 | 6.35 | 0.80 | 4.60 | 1.12 | 2.02 | 3.94 | 1.45 | <20 | 19.45 | 156 | male | 53 | 94.81 | 124.42 | 10.89 |
| Control-075 | 6.22 | 0.80 | 3.67 | 1.15 | 4.58 | 7.81 | 2.81 | <20 | 26.52 | 158 | female | 61 | 67.00 | 131.72 | 11.27 |
| Control-076 | 5.85 | 0.80 | 5.10 | 1.29 | 2.31 | 4.16 | 0.89 | <20 | 26.86 | 155 | female | 47 | 95.08 | 146.03 | 11.10 |
| Control-077 | 6.89 | 0.80 | 3.65 | 1.54 | 2.80 | 5.06 | 1.07 | <20 | 21.45 | 154 | female | 47 | 52.73 | 137.12 | 17.29 |
| Control-078 | 6.90 | 0.80 | 5.30 | 0.76 | 2.30 | 4.49 | 2.65 | <20 | 21.90 | 142 | male | 55 | 73.90 | 132.84 | 14.06 |
| Control-079 | 5.43 | 0.80 | 3.19 | 2.27 | 3.19 | 6.51 | 1.43 | <20 | 27.88 | 178 | female | 50 | 66.42 | 124.65 | 14.68 |
| Control-080 | 4.81 | 0.30 | 5.67 | 1.09 | 2.08 | 4.05 | 0.97 | <20 | 23.37 | 174 | female | 43 | 77.60 | 146.05 | 13.98 |
| **HUA group** | | | | | | | | | | | | | | | |
| HUA-001 | 7.05 | 0.68 | 6.82 | 2.37 | 2.08 | 3.25 | 1.39 | <20 | 26.84 | 492 | male | 44 | 76.65 | 131.37 | 18.14 |
| HUA-002 | 4.80 | 0.87 | 5.34 | 0.91 | 2.59 | 5.44 | 4.33 | <20 | 24.12 | 390 | male | 22 | 86.02 | 121.45 | 16.63 |
| HUA-003 | 6.69 | 1.25 | 6.21 | 1.40 | 1.95 | 5.19 | 1.09 | <20 | 27.89 | 465 | male | 24 | 108.69 | 147.44 | 16.27 |
| HUA-004 | 5.21 | 1.44 | 4.91 | 1.14 | 1.86 | 4.23 | 3.36 | <20 | 25.48 | 569 | male | 71 | 58.09 | 141.81 | 16.92 |
| HUA-005 | 5.38 | 1.09 | 5.06 | 1.10 | 3.61 | 5.54 | 2.52 | <20 | 26.53 | 537 | female | 36 | 86.87 | 144.09 | 16.11 |
| HUA-006 | 6.67 | 0.71 | 2.83 | 2.23 | 1.76 | 3.39 | 2.90 | <20 | 26.57 | 352 | female | 54 | 92.25 | 129.62 | 16.44 |
| HUA-007 | 3.79 | 1.54 | 5.60 | 1.00 | 2.45 | 4.22 | 1.04 | <20 | 27.51 | 479 | female | 68 | 67.10 | 130.45 | 16.38 |
| HUA-008 | 4.65 | 0.31 | 2.40 | 1.03 | 4.23 | 6.60 | 3.23 | <20 | 25.02 | 431 | male | 21 | 75.93 | 123.12 | 17.51 |
| HUA-009 | 5.76 | 0.84 | 3.04 | 1.45 | 3.25 | 5.76 | 1.49 | <20 | 25.75 | 381 | female | 69 | 108.12 | 130.20 | 19.89 |
| HUA-010 | 5.38 | 1.43 | 4.04 | 1.42 | 3.13 | 5.52 | 1.48 | <20 | 24.71 | 390 | female | 52 | 90.29 | 139.30 | 20.06 |
| HUA-011 | 7.02 | 0.90 | 4.25 | 0.89 | 2.89 | 5.02 | 2.62 | <20 | 27.16 | 393 | female | 67 | 100.59 | 139.52 | 17.11 |
| HUA-012 | 5.38 | 1.24 | 3.70 | 2.48 | 2.90 | 6.11 | 3.92 | <20 | 26.08 | 471 | male | 44 | 62.14 | 122.20 | 16.74 |
| HUA-013 | 7.33 | 0.85 | 5.69 | 2.15 | 3.68 | 5.83 | 1.37 | <20 | 25.34 | 428 | female | 69 | 72.88 | 143.98 | 20.41 |
| HUA-014 | 5.47 | 0.86 | 5.92 | 1.76 | 1.87 | 4.20 | 1.05 | <20 | 27.44 | 507 | male | 28 | 68.81 | 142.15 | 18.72 |
| HUA-015 | 4.23 | 0.87 | 5.29 | 1.30 | 3.48 | 4.87 | 3.16 | <20 | 26.52 | 420 | male | 34 | 86.24 | 126.09 | 20.27 |
| HUA-016 | 3.91 | 1.33 | 6.34 | 1.27 | 1.74 | 3.60 | 0.79 | <20 | 26.93 | 511 | male | 15 | 82.88 | 124.22 | 20.80 |
| HUA-017 | 5.22 | 1.59 | 4.87 | 0.94 | 2.82 | 4.23 | 0.91 | <20 | 24.49 | 454 | male | 35 | 56.06 | 125.11 | 16.60 |
| HUA-018 | 6.27 | 0.84 | 4.99 | 0.99 | 2.71 | 4.64 | 2.11 | <20 | 25.79 | 360 | female | 65 | 87.45 | 140.99 | 19.23 |
| HUA-019 | 6.02 | 1.43 | 4.43 | 1.59 | 2.82 | 5.01 | 1.04 | <20 | 26.65 | 481 | male | 28 | 103.22 | 133.15 | 17.52 |
| HUA-020 | 4.30 | 1.10 | 5.27 | 1.82 | 1.75 | 3.32 | 3.15 | <20 | 25.28 | 505 | male | 25 | 62.24 | 128.46 | 20.94 |
| HUA-021 | 5.54 | 0.61 | 4.52 | 1.02 | 2.95 | 5.49 | 4.69 | <20 | 25.12 | 557 | male | 37 | 59.51 | 127.54 | 18.32 |
| HUA-022 | 6.36 | 0.92 | 6.60 | 0.94 | 2.26 | 4.53 | 1.58 | <20 | 26.56 | 513 | male | 27 | 106.35 | 143.44 | 16.92 |
| HUA-023 | 5.61 | 1.44 | 5.04 | 2.22 | 1.91 | 4.11 | 3.29 | <20 | 26.38 | 413 | female | 57 | 69.22 | 134.65 | 17.53 |
| HUA-024 | 4.26 | 0.53 | 7.73 | 2.30 | 2.35 | 6.53 | 4.25 | <20 | 25.46 | 550 | male | 41 | 61.66 | 128.70 | 17.75 |
| HUA-025 | 5.70 | 0.74 | 3.92 | 1.65 | 3.37 | 6.07 | 4.27 | <20 | 25.33 | 501 | male | 24 | 60.16 | 138.13 | 19.20 |
| HUA-026 | 5.41 | 1.14 | 5.54 | 1.37 | 2.25 | 4.08 | 4.55 | <20 | 27.77 | 494 | male | 26 | 105.17 | 133.51 | 16.42 |
| HUA-027 | 4.97 | 1.24 | 4.71 | 1.21 | 3.73 | 5.57 | 1.12 | <20 | 24.91 | 519 | male | 44 | 77.71 | 132.26 | 18.15 |
| HUA-028 | 5.71 | 1.60 | 5.10 | 1.64 | 3.36 | 5.93 | 3.89 | <20 | 25.65 | 452 | female | 30 | 73.26 | 133.21 | 18.31 |
| HUA-029 | 6.53 | 1.46 | 3.33 | 1.95 | 1.71 | 4.67 | 3.97 | <20 | 24.64 | 601 | male | 52 | 57.53 | 122.18 | 18.58 |
| HUA-030 | 6.59 | 0.80 | 2.80 | 1.84 | 1.84 | 6.83 | 2.07 | <20 | 25.66 | 547 | male | 59 | 109.19 | 145.57 | 16.66 |
| HUA-031 | 6.70 | 1.60 | 7.62 | 0.75 | 2.74 | 4.30 | 3.08 | <20 | 25.77 | 551 | male | 31 | 76.52 | 138.15 | 20.69 |
| HUA-032 | 5.63 | 0.27 | 4.62 | 1.15 | 2.13 | 6.00 | 2.88 | <20 | 24.55 | 480 | male | 55 | 57.46 | 132.48 | 20.13 |
| HUA-033 | 5.72 | 1.38 | 6.36 | 1.96 | 2.82 | 4.02 | 2.54 | <20 | 24.32 | 476 | male | 66 | 66.66 | 137.74 | 17.85 |
| HUA-034 | 4.41 | 1.49 | 5.16 | 1.19 | 2.77 | 3.57 | 1.07 | <20 | 27.33 | 363 | male | 51 | 65.97 | 135.42 | 19.96 |
| HUA-035 | 5.84 | 0.64 | 4.13 | 2.33 | 1.97 | 4.51 | 1.02 | <20 | 25.84 | 520 | male | 35 | 95.24 | 134.95 | 19.50 |
| HUA-036 | 6.76 | 0.31 | 6.73 | 1.22 | 2.29 | 4.54 | 1.29 | <20 | 25.22 | 586 | male | 55 | 81.74 | 140.45 | 17.36 |
| HUA-037 | 6.93 | 0.64 | 2.36 | 1.57 | 1.95 | 3.65 | 4.02 | <20 | 27.18 | 475 | male | 54 | 103.69 | 145.22 | 18.19 |
| HUA-038 | 5.17 | 1.05 | 2.92 | 2.07 | 2.48 | 3.38 | 3.64 | <20 | 26.12 | 569 | male | 63 | 94.58 | 128.24 | 18.41 |
| HUA-039 | 6.25 | 0.25 | 6.14 | 1.29 | 2.36 | 4.62 | 1.80 | <20 | 26.67 | 546 | male | 37 | 94.99 | 134.33 | 17.98 |
| HUA-040 | 6.24 | 1.03 | 2.86 | 2.33 | 2.64 | 5.96 | 2.40 | <20 | 27.27 | 412 | male | 62 | 100.91 | 126.88 | 16.47 |
| HUA-041 | 6.60 | 0.76 | 4.56 | 1.89 | 1.98 | 3.84 | 4.35 | <20 | 27.33 | 480 | female | 64 | 108.41 | 129.83 | 17.38 |
| HUA-042 | 4.73 | 1.04 | 3.66 | 2.12 | 2.64 | 3.53 | 3.30 | <20 | 25.74 | 461 | female | 35 | 75.43 | 133.67 | 17.88 |
| HUA-043 | 4.82 | 0.23 | 3.47 | 1.46 | 2.12 | 5.39 | 1.14 | <20 | 26.80 | 483 | male | 39 | 97.48 | 133.33 | 18.84 |
| HUA-044 | 5.62 | 0.21 | 6.62 | 0.90 | 2.74 | 4.00 | 2.13 | <20 | 27.06 | 532 | male | 61 | 84.09 | 141.23 | 16.40 |
| HUA-045 | 4.34 | 0.49 | 5.11 | 2.04 | 2.70 | 6.76 | 1.00 | <20 | 27.57 | 512 | male | 36 | 77.00 | 126.07 | 19.93 |
| HUA-046 | 6.60 | 0.88 | 5.35 | 1.71 | 2.83 | 5.28 | 1.88 | <20 | 24.92 | 433 | female | 60 | 101.98 | 127.48 | 16.58 |
| HUA-047 | 5.24 | 0.22 | 4.91 | 2.41 | 1.97 | 5.95 | 1.38 | <20 | 25.64 | 495 | male | 65 | 63.03 | 132.51 | 18.70 |
| HUA-048 | 6.23 | 0.94 | 4.74 | 1.49 | 2.77 | 3.69 | 4.10 | <20 | 27.50 | 487 | male | 40 | 106.48 | 123.60 | 17.71 |
| HUA-049 | 6.67 | 1.45 | 6.58 | 2.30 | 2.37 | 4.18 | 3.64 | <20 | 25.81 | 459 | female | 43 | 99.95 | 127.03 | 16.29 |
| HUA-050 | 5.20 | 1.36 | 2.94 | 2.17 | 2.20 | 3.86 | 4.10 | <20 | 27.65 | 481 | male | 62 | 62.27 | 125.34 | 16.44 |
| HUA-051 | 5.06 | 1.27 | 5.91 | 1.94 | 2.03 | 6.70 | 1.89 | <20 | 25.56 | 418 | female | 53 | 82.39 | 127.80 | 16.23 |
| HUA-052 | 5.89 | 1.26 | 6.36 | 1.60 | 2.04 | 3.43 | 2.65 | <20 | 26.25 | 498 | male | 35 | 68.47 | 121.64 | 16.57 |
| HUA-053 | 4.24 | 0.61 | 6.03 | 1.79 | 2.90 | 5.22 | 3.46 | <20 | 25.83 | 490 | male | 29 | 74.16 | 131.80 | 18.87 |
| HUA-054 | 6.19 | 0.66 | 7.88 | 2.10 | 2.54 | 5.55 | 4.03 | <20 | 27.63 | 541 | male | 54 | 96.89 | 139.52 | 16.20 |
| HUA-055 | 6.69 | 0.44 | 6.07 | 2.38 | 1.75 | 3.49 | 3.53 | <20 | 24.91 | 586 | male | 54 | 73.48 | 127.73 | 19.27 |
| HUA-056 | 6.53 | 0.54 | 7.43 | 1.12 | 2.79 | 5.07 | 3.68 | <20 | 25.10 | 503 | male | 66 | 63.82 | 126.96 | 19.84 |
| HUA-057 | 5.99 | 1.26 | 2.58 | 0.86 | 1.91 | 4.45 | 4.12 | <20 | 27.25 | 452 | male | 29 | 93.86 | 139.18 | 17.61 |
| HUA-058 | 5.73 | 0.47 | 4.39 | 0.92 | 2.83 | 4.34 | 0.96 | <20 | 24.31 | 598 | male | 28 | 98.71 | 131.12 | 16.26 |
| HUA-059 | 6.81 | 1.41 | 5.69 | 0.91 | 2.50 | 5.59 | 1.19 | <20 | 27.58 | 399 | male | 68 | 74.30 | 123.74 | 19.50 |
| HUA-060 | 6.70 | 0.44 | 5.24 | 1.64 | 2.08 | 6.31 | 4.27 | <20 | 25.84 | 433 | female | 64 | 82.71 | 137.18 | 19.14 |
| HUA-061 | 6.52 | 0.93 | 3.96 | 2.07 | 1.73 | 4.33 | 3.10 | <20 | 26.74 | 515 | male | 64 | 66.19 | 128.34 | 19.84 |
| HUA-062 | 5.42 | 0.79 | 7.36 | 2.03 | 2.25 | 5.29 | 1.09 | <20 | 27.47 | 486 | male | 54 | 83.64 | 140.14 | 19.44 |
| **AGA group** | | | | | | | | | | | | | | | |
| AGA-001 | 4.23 | 37.10 | 5.08 | 0.70 | 1.90 | 3.48 | 2.01 | <20 | 29.56 | 383 | male | 66 | 99.32 | 129.56 | 26.84 |
| AGA-002 | 5.78 | 62.60 | 2.09 | 1.63 | 1.49 | 3.87 | 1.23 | <20 | 28.01 | 404 | male | 31 | 96.12 | 134.47 | 19.04 |
| AGA-003 | 12.05 | 16.40 | 5.45 | 0.84 | 3.39 | 5.26 | 2.32 | <20 | 26.57 | 569 | male | 60 | 126.40 | 137.63 | 35.52 |
| AGA-004 | 12.52 | 10.98 | 3.79 | 0.80 | 2.98 | 4.56 | 2.08 | <20 | 26.20 | 465 | male | 35 | 126.78 | 131.03 | 32.77 |
| AGA-005 | 7.44 | 6.60 | 3.01 | 1.11 | 3.22 | 4.90 | 2.34 | <20 | 25.77 | 520 | male | 33 | 119.76 | 125.98 | 43.39 |
| AGA-006 | 7.66 | 69.02 | 4.46 | 0.87 | 2.92 | 4.90 | 1.87 | <20 | 30.97 | 590 | male | 49 | 75.99 | 123.06 | 35.31 |
| AGA-007 | 5.47 | 8.10 | 5.50 | 1.27 | 3.05 | 5.25 | 2.17 | <20 | 30.01 | 480 | male | 69 | 93.73 | 131.65 | 21.81 |
| AGA-008 | 12.25 | 53.50 | 5.16 | 0.98 | 3.66 | 5.78 | 3.22 | <20 | 25.78 | 201 | male | 30 | 88.00 | 129.86 | 34.99 |
| AGA-009 | 11.36 | 20.20 | 4.50 | 0.80 | 3.22 | 4.71 | 1.42 | <20 | 30.89 | 391 | male | 70 | 76.65 | 138.86 | 43.38 |
| AGA-010 | 5.51 | 4.40 | 8.00 | 1.45 | 2.19 | 4.50 | 1.21 | 23.9 | 27.41 | 517 | male | 81 | 88.99 | 144.14 | 42.95 |
| AGA-011 | 8.32 | 6.70 | 4.38 | 1.15 | 3.88 | 5.64 | 0.93 | <20 | 30.86 | 475 | male | 48 | 79.09 | 140.92 | 40.05 |
| AGA-012 | 4.48 | 44.80 | 6.59 | 0.95 | 3.00 | 4.70 | 1.34 | <20 | 26.59 | 430 | male | 61 | 77.10 | 147.71 | 25.44 |
| AGA-013 | 10.07 | 8.00 | 4.81 | 1.01 | 2.96 | 4.60 | 1.67 | <20 | 31.12 | 512 | male | 36 | 122.54 | 124.24 | 16.50 |
| AGA-014 | 11.47 | 62.70 | 4.12 | 1.41 | 3.36 | 5.53 | 1.79 | <20 | 27.01 | 348 | male | 45 | 119.05 | 120.31 | 26.77 |
| AGA-015 | 7.96 | 38.45 | 4.63 | 1.03 | 3.22 | 4.96 | 2.31 | <20 | 31.66 | 472 | male | 30 | 118.26 | 127.62 | 31.25 |
| AGA-016 | 5.78 | 1.90 | 5.59 | 1.02 | 4.40 | 5.99 | 1.12 | <20 | 29.42 | 359 | female | 54 | 77.34 | 122.19 | 22.86 |
| AGA-017 | 12.61 | 19.40 | 4.65 | 1.13 | 2.13 | 4.16 | 2.41 | <20 | 28.73 | 351 | male | 30 | 128.98 | 132.31 | 31.87 |
| AGA-018 | 13.40 | 15.80 | 5.25 | 1.40 | 1.94 | 3.83 | 0.70 | <20 | 29.67 | 360 | male | 61 | 121.97 | 138.94 | 33.93 |
| AGA-019 | 6.56 | 8.60 | 5.32 | 0.94 | 3.22 | 5.28 | 2.60 | <20 | 28.14 | 394 | male | 63 | 128.20 | 148.52 | 40.40 |
| AGA-020 | 6.65 | 19.40 | 6.70 | 0.75 | 1.82 | 3.40 | 1.75 | <20 | 27.85 | 595 | male | 62 | 83.10 | 148.50 | 26.45 |
| AGA-021 | 6.59 | 30.27 | 5.40 | 1.08 | 3.39 | 4.84 | 3.34 | <20 | 29.45 | 268 | male | 66 | 114.22 | 123.21 | 27.80 |
| AGA-022 | 8.30 | 13.15 | 5.20 | 1.23 | 4.52 | 6.71 | 1.89 | <20 | 29.86 | 276 | male | 51 | 102.01 | 141.98 | 25.16 |
| AGA-023 | 3.96 | 34.30 | 3.20 | 1.28 | 3.18 | 5.75 | 2.38 | <20 | 29.05 | 540 | male | 35 | 88.45 | 127.26 | 39.69 |
| AGA-024 | 9.01 | 30.20 | 3.35 | 1.06 | 3.42 | 5.29 | 1.72 | <20 | 26.75 | 513 | male | 35 | 92.15 | 132.43 | 19.47 |
| AGA-025 | 5.35 | 13.20 | 6.32 | 0.89 | 1.36 | 2.89 | 1.13 | <20 | 28.25 | 429 | male | 65 | 83.87 | 143.48 | 37.81 |
| AGA-026 | 13.06 | 13.50 | 8.48 | 1.45 | 3.14 | 5.36 | 1.37 | <20 | 30.40 | 594 | female | 62 | 98.96 | 133.71 | 27.14 |
| AGA-027 | 4.84 | 2.30 | 5.46 | 0.89 | 4.31 | 4.70 | 1.89 | <20 | 29.45 | 444 | male | 66 | 78.20 | 144.46 | 33.54 |
| AGA-028 | 6.73 | 9.40 | 4.69 | 0.71 | 2.91 | 4.25 | 1.28 | <20 | 30.85 | 553 | male | 47 | 122.81 | 143.23 | 15.80 |
| AGA-029 | 7.79 | 3.60 | 3.57 | 1.12 | 3.62 | 5.79 | 2.23 | <20 | 30.61 | 475 | male | 63 | 92.26 | 123.50 | 32.37 |
| AGA-030 | 5.11 | 7.10 | 5.51 | 0.68 | 2.92 | 4.59 | 3.44 | <20 | 29.78 | 533 | male | 31 | 120.67 | 147.00 | 37.56 |
| AGA-031 | 5.96 | 21.56 | 3.08 | 0.99 | 1.88 | 3.22 | 1.05 | <20 | 31.88 | 340 | male | 21 | 105.90 | 145.01 | 28.92 |
| AGA-032 | 10.30 | 35.12 | 7.62 | 0.99 | 1.11 | 4.50 | 1.96 | <20 | 29.21 | 547 | male | 31 | 127.95 | 145.23 | 24.29 |
| AGA-033 | 6.72 | 8.40 | 2.69 | 0.96 | 3.82 | 5.93 | 1.72 | <20 | 30.09 | 505 | male | 29 | 98.56 | 145.12 | 39.66 |
| AGA-034 | 8.65 | 64.60 | 4.99 | 1.62 | 2.34 | 4.48 | 1.01 | <20 | 28.08 | 545 | male | 27 | 129.17 | 146.41 | 40.04 |
| AGA-035 | 11.41 | 31.80 | 4.35 | 1.08 | 2.33 | 3.87 | 1.21 | <20 | 29.49 | 447 | male | 55 | 77.59 | 137.51 | 40.13 |
| AGA-036 | 15.41 | 7.20 | 4.92 | 1.01 | 1.36 | 4.85 | 1.90 | <20 | 31.13 | 599 | male | 29 | 96.59 | 134.70 | 42.06 |
| AGA-037 | 9.56 | 70.00 | 4.07 | 0.84 | 3.06 | 5.17 | 3.80 | <20 | 28.10 | 662 | male | 40 | 75.60 | 146.05 | 28.44 |
| AGA-038 | 7.37 | 81.60 | 7.03 | 1.38 | 3.10 | 5.30 | 1.18 | <20 | 26.11 | 743 | male | 27 | 112.08 | 130.63 | 32.08 |
| AGA-039 | 11.41 | 17.37 | 5.76 | 0.87 | 2.53 | 3.85 | 1.22 | <20 | 28.69 | 588 | female | 79 | 101.18 | 137.23 | 16.47 |
| AGA-040 | 6.87 | 41.00 | 5.57 | 1.03 | 2.98 | 4.45 | 1.98 | <20 | 27.41 | 542 | male | 32 | 101.78 | 127.69 | 28.71 |
| AGA-041 | 7.25 | 4.40 | 4.01 | 1.05 | 4.31 | 7.16 | 3.85 | <20 | 25.89 | 307 | male | 35 | 116.75 | 146.80 | 40.64 |
| AGA-042 | 4.41 | 2.30 | 6.77 | 1.48 | 1.94 | 5.84 | 4.97 | <20 | 30.81 | 648 | male | 65 | 86.04 | 137.77 | 24.17 |
| AGA-043 | 8.24 | 8.80 | 7.03 | 1.38 | 5.55 | 7.90 | 1.12 | <20 | 31.07 | 263 | female | 71 | 112.32 | 136.18 | 34.98 |
| AGA-044 | 4.76 | 34.70 | 27.33 | 0.83 | 1.26 | 2.34 | 0.49 | <20 | 28.47 | 213 | male | 86 | 98.33 | 149.26 | 34.05 |
| AGA-045 | 7.56 | 11.20 | 9.87 | 1.06 | 3.30 | 4.90 | 1.99 | <20 | 28.30 | 562 | male | 29 | 109.08 | 130.03 | 29.37 |
| AGA-046 | 8.20 | 71.40 | 2.80 | 1.37 | 1.11 | 3.09 | 1.33 | <20 | 25.85 | 346 | male | 57 | 88.13 | 135.56 | 44.38 |
| AGA-047 | 9.45 | 49.70 | 5.58 | 1.13 | 2.68 | 4.90 | 2.21 | <20 | 26.61 | 452 | male | 55 | 89.44 | 138.30 | 31.19 |
| AGA-048 | 7.28 | 15.40 | 10.82 | 1.06 | 2.54 | 4.39 | 0.98 | <20 | 27.96 | 307 | male | 76 | 109.14 | 131.56 | 38.67 |
| AGA-049 | 5.05 | 3.20 | 7.26 | 1.53 | 2.45 | 4.80 | 1.39 | 21.2 | 30.57 | 620 | male | 60 | 99.86 | 141.71 | 29.55 |
| AGA-050 | 9.14 | 22.70 | 5.07 | 0.90 | 2.20 | 4.91 | 4.70 | <20 | 29.83 | 519 | male | 44 | 114.60 | 126.84 | 16.55 |
| AGA-051 | 8.81 | 12.90 | 6.63 | 1.08 | 3.30 | 5.41 | 1.86 | 26 | 26.93 | 631 | male | 52 | 87.93 | 147.25 | 44.13 |
| AGA-052 | 13.41 | 8.50 | 2.98 | 1.00 | 2.68 | 4.44 | 2.00 | <20 | 31.72 | 488 | male | 25 | 90.46 | 127.35 | 26.33 |
| AGA-053 | 7.54 | 64.80 | 7.40 | 1.41 | 2.57 | 4.41 | 0.54 | <20 | 30.48 | 251 | male | 60 | 83.74 | 132.43 | 35.72 |
| AGA-054 | 4.70 | 16.70 | 6.81 | 1.01 | 2.78 | 4.80 | 2.20 | <20 | 31.75 | 529 | male | 91 | 109.83 | 137.51 | 21.10 |
| AGA-055 | 9.10 | 78.63 | 3.09 | 0.88 | 2.68 | 4.36 | 2.00 | <20 | 26.64 | 688 | male | 28 | 90.56 | 124.31 | 33.69 |
| AGA-056 | 10.17 | 4.40 | 3.65 | 1.03 | 2.78 | 4.45 | 1.66 | <20 | 30.58 | 486 | male | 33 | 127.39 | 120.36 | 28.82 |
| AGA-057 | 14.45 | 79.90 | 20.62 | 1.57 | 1.74 | 3.88 | 0.82 | <20 | 31.20 | 639 | male | 52 | 76.56 | 141.44 | 20.12 |
| AGA-058 | 10.35 | 28.50 | 3.63 | 1.18 | 3.76 | 4.95 | 2.09 | <20 | 26.11 | 566 | male | 55 | 81.74 | 128.66 | 34.27 |
| AGA-059 | 9.36 | 19.10 | 3.98 | 1.11 | 3.05 | 5.55 | 1.97 | <20 | 27.77 | 553 | male | 25 | 111.32 | 133.81 | 17.27 |
| AGA-060 | 10.50 | 62.03 | 3.75 | 1.10 | 3.94 | 5.70 | 1.25 | <20 | 27.83 | 440 | male | 32 | 118.15 | 135.53 | 37.66 |
| AGA-061 | 5.55 | 2.50 | 3.69 | 1.16 | 3.93 | 6.11 | 2.52 | <20 | 30.74 | / | male | 56 | 103.57 | 124.21 | 31.32 |
| AGA-062 | 7.24 | 51.26 | 4.73 | 1.20 | 2.71 | 4.38 | 1.75 | <20 | 29.75 | 572 | male | 20 | 125.97 | 138.76 | 15.00 |
| AGA-063 | 11.16 | 70.17 | 6.33 | 1.03 | 3.76 | 6.18 | 3.64 | <20 | 25.61 | 450 | male | 64 | 114.69 | 120.52 | 42.40 |
| AGA-064 | 9.65 | 78.10 | 6.11 | 1.25 | 3.30 | 4.93 | 1.42 | <20 | 31.58 | 262 | male | 63 | 111.32 | 136.95 | 30.98 |
| AGA-065 | 8.00 | 42.30 | 4.23 | 0.92 | 2.94 | 4.73 | 2.17 | <20 | 30.69 | 514 | male | 34 | 97.07 | 123.19 | 21.85 |
| AGA-066 | 7.30 | 57.00 | 6.62 | 1.72 | 2.86 | 5.49 | 1.80 | <20 | 29.87 | 462 | female | 70 | 94.04 | 148.32 | 30.88 |
| AGA-067 | 7.92 | 123.40 | 4.86 | 0.87 | 2.98 | 4.66 | 2.07 | <20 | 31.61 | 371 | male | 55 | 119.64 | 147.35 | 23.98 |
| AGA-068 | 6.59 | 1.30 | 7.75 | 0.87 | 2.50 | 4.48 | 2.65 | <20 | 26.21 | 527 | male | 65 | 86.40 | 141.23 | 31.43 |
| AGA-069 | 12.33 | 29.05 | 9.89 | 1.00 | 3.94 | 4.99 | 2.14 | <20 | 26.25 | 542 | male | 56 | 112.40 | 139.84 | 31.56 |
| **GIP group** | | | | | | | | | | | | | | | |
| GIP-001 | 4.89 | 2.48 | 5.15 | 0.96 | 2.40 | 8.10 | 6.94 | <20 | 26.34 | 419 | male | 36 | 86.69 | 125.66 | 14.28 |
| GIP-002 | 7.39 | 0.20 | 3.37 | 1.10 | 3.20 | 5.77 | 2.21 | <20 | 25.85 | 519 | male | 31 | 88.88 | 140.49 | 14.49 |
| GIP-003 | 8.08 | 0.58 | 6.52 | 0.90 | 2.80 | 6.09 | 2.18 | <20 | 27.80 | 376 | male | 67 | 129.86 | 135.55 | 19.48 |
| GIP-004 | 5.55 | 4.84 | 3.91 | 1.06 | 2.70 | 5.61 | 3.91 | <20 | 24.64 | 373 | male | 42 | 101.89 | 128.06 | 17.96 |
| GIP-005 | 8.54 | 3.34 | 4.11 | 0.87 | 3.80 | 5.70 | 2.71 | <20 | 25.44 | 339 | male | 33 | 77.25 | 132.47 | 16.58 |
| GIP-006 | 6.57 | 96.50 | 5.92 | 1.10 | 3.30 | 4.11 | 0.93 | <20 | 22.57 | 381 | female | 64 | 87.91 | 129.15 | 16.58 |
| GIP-007 | 8.78 | 1.63 | 4.16 | 1.03 | 2.23 | 5.10 | 4.58 | <20 | 24.04 | 455 | male | 46 | 88.92 | 132.48 | 16.71 |
| GIP-008 | 6.30 | 1.69 | 4.23 | 1.06 | 4.02 | 5.77 | 1.22 | <20 | 23.56 | 420 | male | 42 | 89.74 | 122.56 | 18.64 |
| GIP-009 | 6.61 | 4.62 | 5.68 | 1.46 | 4.26 | 6.66 | 1.80 | <20 | 26.92 | 355 | male | 36 | 93.85 | 125.63 | 15.43 |
| GIP-010 | 5.24 | 2.09 | 6.90 | 1.33 | 2.57 | 4.66 | 1.78 | <20 | 25.39 | 412 | male | 69 | 100.82 | 147.89 | 18.58 |
| GIP-011 | 4.78 | 0.59 | 3.65 | 0.86 | 2.51 | 5.02 | 4.13 | <20 | 26.68 | 434 | male | 40 | 85.23 | 144.15 | 16.31 |
| GIP-012 | 7.36 | 120.80 | 5.27 | 0.90 | 2.70 | 6.06 | 2.77 | <20 | 23.01 | 396 | male | 40 | 116.59 | 127.83 | 15.43 |
| GIP-013 | 9.00 | 0.92 | 3.64 | 1.10 | 3.10 | 4.97 | 1.22 | <20 | 27.43 | 364 | male | 34 | 122.26 | 136.54 | 19.28 |
| GIP-014 | 5.79 | 8.40 | 6.78 | 1.05 | 2.54 | 5.06 | 2.18 | <20 | 25.99 | 531 | female | 65 | 118.16 | 134.61 | 16.24 |
| GIP-015 | 8.11 | 3.79 | 7.62 | 0.90 | 2.90 | 3.77 | 1.38 | <20 | 26.68 | / | male | 35 | 115.60 | 134.31 | 14.99 |
| GIP-016 | 5.42 | 0.19 | 4.93 | 1.18 | 2.17 | 4.06 | 1.00 | <20 | 25.30 | 354 | male | 23 | 113.22 | 128.58 | 16.35 |
| GIP-017 | 10.92 | 3.97 | 5.10 | 1.10 | 3.01 | 4.67 | 3.52 | <20 | 27.70 | 625 | male | 23 | 111.18 | 144.76 | 14.43 |
| GIP-018 | 9.88 | 3.42 | 4.65 | 1.16 | 3.04 | 4.87 | 1.23 | <20 | 22.93 | 540 | male | 38 | 119.90 | 120.14 | 14.14 |
| GIP-019 | 7.18 | 2.86 | 3.77 | 0.90 | 3.05 | 4.75 | 4.35 | <20 | 24.26 | 654 | male | 20 | 106.98 | 129.83 | 18.48 |
| GIP-020 | 5.34 | 3.78 | 4.51 | 1.18 | 4.35 | 6.52 | 1.92 | <20 | 23.91 | 288 | male | 53 | 106.16 | 130.34 | 16.66 |
| GIP-021 | 8.85 | 0.50 | 3.68 | 1.17 | 4.09 | 6.63 | 3.19 | <20 | 23.02 | 375 | male | 48 | 77.71 | 129.46 | 18.76 |
| GIP-022 | 9.49 | 9.10 | 4.83 | 1.22 | 3.07 | 4.75 | 0.68 | <20 | 25.53 | 547 | male | 35 | 109.65 | 120.59 | 14.89 |
| GIP-023 | 6.18 | 2.10 | 4.48 | 1.35 | 2.91 | 4.97 | 1.06 | <20 | 24.32 | 419 | male | 82 | 117.13 | 133.26 | 14.04 |
| GIP-024 | 5.01 | 15.10 | 4.63 | 0.69 | 1.82 | 3.17 | 1.98 | <20 | 25.59 | 529 | male | 57 | 87.45 | 146.02 | 17.79 |
| GIP-025 | 5.34 | 4.01 | 3.09 | 0.69 | 1.95 | 4.75 | 3.52 | <20 | 23.55 | 613 | male | 23 | 87.39 | 129.83 | 15.95 |
| GIP-026 | 8.71 | 0.68 | 4.15 | 0.81 | 2.87 | 4.66 | 2.32 | <20 | 24.68 | 255 | male | 32 | 110.77 | 123.68 | 16.12 |
| GIP-027 | 8.01 | 4.87 | 3.40 | 1.13 | 3.68 | 5.57 | 1.75 | <20 | 24.14 | 700 | male | 40 | 129.13 | 133.34 | 17.54 |
| GIP-028 | 6.85 | 3.24 | 5.81 | 1.10 | 3.60 | 5.64 | 0.93 | <20 | 24.01 | 543 | male | 45 | 108.72 | 123.72 | 17.27 |
| GIP-029 | 8.05 | 1.17 | 3.79 | 0.90 | 2.40 | 4.93 | 4.56 | <20 | 25.54 | 433 | male | 32 | 80.10 | 124.44 | 14.83 |
| GIP-030 | 4.65 | 2.02 | 2.99 | 1.10 | 3.11 | 3.67 | 1.23 | <20 | 27.12 | 359 | male | 25 | 111.93 | 120.68 | 15.40 |
| GIP-031 | 9.02 | 0.32 | 4.68 | 0.90 | 3.80 | 5.64 | 1.47 | <20 | 27.79 | 297 | male | 32 | 101.57 | 143.18 | 14.04 |
| GIP-032 | 5.59 | 4.64 | 7.16 | 0.93 | 2.66 | 5.07 | 3.58 | <20 | 23.66 | 365 | male | 39 | 97.96 | 142.11 | 17.18 |
| GIP-033 | 8.87 | 5.60 | 4.23 | 1.10 | 1.89 | 4.56 | 1.22 | <20 | 24.36 | 338 | male | 33 | 98.95 | 121.58 | 17.48 |
| GIP-034 | 4.75 | 4.31 | 8.04 | 1.00 | 2.49 | 7.89 | 0.93 | <20 | 24.97 | 272 | male | 64 | 129.97 | 135.22 | 17.65 |
| GIP-035 | 10.71 | 4.68 | 5.23 | 0.78 | 2.67 | 5.00 | 4.35 | <20 | 26.36 | 354 | male | 39 | 77.88 | 138.18 | 13.46 |
| GIP-036 | 8.58 | 1.84 | 6.33 | 0.98 | 2.96 | 4.51 | 1.47 | <20 | 25.73 | 444 | male | 28 | 94.19 | 136.08 | 18.83 |
| GIP-037 | 6.66 | 0.54 | 6.26 | 0.97 | 3.89 | 5.98 | 2.04 | <20 | 26.51 | 247 | male | 55 | 105.06 | 122.96 | 15.94 |
| GIP-038 | 10.40 | 4.66 | 5.10 | 1.00 | 3.32 | 4.93 | 2.21 | <20 | 24.58 | 279 | male | 34 | 98.42 | 132.22 | 19.76 |
| GIP-039 | 8.68 | 0.30 | 5.10 | 1.10 | 3.23 | 6.18 | 2.18 | <20 | 26.22 | 478 | male | 40 | 95.01 | 121.28 | 18.53 |
| GIP-040 | 10.46 | 2.70 | 8.86 | 0.90 | 2.77 | 3.34 | 2.77 | <20 | 25.71 | 365 | female | 56 | 129.44 | 148.17 | 19.74 |
| GIP-041 | 5.82 | 21.50 | 5.49 | 1.26 | 3.19 | 4.91 | 1.30 | <20 | 24.91 | 430 | male | 50 | 104.05 | 145.53 | 17.91 |
| GIP-042 | 5.43 | 2.16 | 4.17 | 1.01 | 3.45 | 5.19 | 2.00 | <20 | 27.24 | 393 | male | 23 | 94.47 | 122.20 | 15.85 |
| GIP-043 | 7.34 | 1.00 | 4.55 | 1.90 | 2.44 | 5.62 | 2.12 | <20 | 23.59 | 458 | male | 66 | 95.47 | 140.15 | 14.47 |
| GIP-044 | 10.34 | 2.65 | 4.21 | 1.00 | 2.68 | 5.88 | 2.58 | <20 | 23.18 | 398 | male | 41 | 117.85 | 134.96 | 19.35 |
| GIP-045 | 5.14 | 0.64 | 8.30 | 1.55 | 1.62 | 3.84 | 1.23 | <20 | 26.09 | 422 | male | 81 | 124.56 | 143.46 | 17.22 |
| GIP-046 | 10.13 | 1.26 | 5.76 | 1.10 | 3.01 | 4.93 | 2.34 | <20 | 27.20 | 387 | male | 47 | 115.47 | 143.93 | 17.20 |
| GIP-047 | 9.28 | 3.07 | 3.18 | 1.41 | 3.77 | 5.73 | 0.97 | <20 | 26.84 | 346 | female | 35 | 107.15 | 147.38 | 18.41 |
| GIP-048 | 5.57 | 2.70 | 3.23 | 1.00 | 3.03 | 5.22 | 1.22 | <20 | 25.20 | 446 | male | 41 | 116.89 | 132.33 | 14.93 |
| GIP-049 | 6.79 | 0.97 | 4.64 | 1.19 | 3.31 | 4.99 | 1.09 | <20 | 27.21 | 546 | male | 39 | 94.99 | 134.56 | 19.95 |
| GIP-050 | 5.96 | 2.50 | 3.17 | 0.91 | 1.96 | 3.34 | 1.21 | <20 | 24.44 | 325 | male | 62 | 77.62 | 130.38 | 14.43 |
| GIP-051 | 4.45 | 1.06 | 7.25 | 0.75 | 2.40 | 3.67 | 1.60 | <20 | 25.20 | 585 | male | 23 | 122.76 | 139.93 | 15.52 |
| GIP-052 | 5.88 | 0.87 | 5.64 | 1.00 | 1.96 | 6.13 | 2.21 | <20 | 26.85 | 396 | male | 31 | 83.97 | 135.83 | 16.60 |
| GIP-053 | 7.47 | 4.80 | 3.87 | 1.11 | 2.91 | 5.32 | 4.71 | <20 | 27.58 | 640 | male | 25 | 78.80 | 135.05 | 19.68 |
| GIP-054 | 8.19 | 1.82 | 4.12 | 1.06 | 1.79 | 3.35 | 1.91 | <20 | 24.08 | 364 | male | 57 | 87.94 | 139.28 | 19.38 |
| GIP-055 | 8.06 | 4.52 | 4.63 | 1.00 | 3.19 | 5.61 | 2.21 | <20 | 23.87 | 504 | male | 33 | 105.27 | 131.98 | 14.40 |
| GIP-056 | 7.88 | 4.60 | 5.70 | 0.76 | 2.34 | 4.51 | 2.49 | <20 | 23.21 | 443 | male | 52 | 123.46 | 147.79 | 15.19 |
| GIP-057 | 8.40 | 2.40 | 9.55 | 1.00 | 3.29 | 3.67 | 4.35 | <20 | 26.04 | 298 | male | 62 | 84.97 | 132.63 | 16.67 |
| GIP-058 | 6.45 | 3.31 | 6.24 | 1.02 | 2.71 | 4.11 | 0.93 | <20 | 22.67 | 526 | male | 40 | 111.03 | 125.05 | 16.04 |
| GIP-059 | 10.80 | 4.03 | 4.69 | 0.94 | 3.23 | 5.27 | 3.48 | <20 | 22.50 | 440 | male | 41 | 91.64 | 136.31 | 19.33 |
| GIP-060 | 6.47 | 2.12 | 5.10 | 1.00 | 2.52 | 5.18 | 2.77 | <20 | 23.89 | 274 | male | 65 | 119.10 | 134.83 | 15.01 |
| GIP-061 | 4.31 | 1.30 | 4.85 | 1.14 | 2.23 | 4.36 | 2.05 | <20 | 25.13 | 240 | male | 32 | 125.03 | 131.08 | 18.87 |
| GIP-062 | 6.91 | 29.50 | 5.80 | 1.00 | 3.34 | 5.19 | 2.18 | <20 | 24.13 | 460 | male | 76 | 119.20 | 144.60 | 13.79 |
| GIP-063 | 8.14 | 1.18 | 5.34 | 0.69 | 2.04 | 3.77 | 2.91 | <20 | 24.55 | 435 | male | 71 | 119.27 | 145.47 | 15.79 |
| GIP-064 | 7.23 | 4.66 | 3.81 | 1.06 | 3.76 | 5.21 | 1.86 | <20 | 24.69 | 405 | male | 33 | 129.51 | 140.40 | 17.60 |
| GIP-065 | 6.23 | 4.50 | 3.81 | 1.00 | 2.66 | 5.33 | 1.22 | <20 | 24.33 | 321 | male | 22 | 97.61 | 128.91 | 14.18 |
| GIP-066 | 7.20 | 0.80 | 4.05 | 1.04 | 3.44 | 5.01 | 2.58 | <20 | 23.73 | 395 | male | 53 | 128.71 | 137.70 | 19.54 |
| GIP-067 | 6.46 | 0.80 | 4.35 | 1.13 | 3.70 | 5.59 | 1.67 | <20 | 23.05 | 249 | male | 72 | 95.35 | 132.38 | 17.19 |
| GIP-068 | 7.71 | 1.92 | 4.96 | 0.90 | 2.86 | 10.87 | 10.42 | <20 | 25.26 | 378 | male | 36 | 91.74 | 146.15 | 17.13 |
| GIP-069 | 11.25 | 7.50 | 5.32 | 1.00 | 2.56 | 5.21 | 2.21 | <20 | 25.28 | 573 | male | 35 | 125.33 | 133.40 | 18.92 |
| GIP-070 | 6.67 | 0.90 | 3.56 | 0.76 | 3.47 | 6.18 | 4.47 | <20 | 24.33 | 412 | male | 32 | 96.64 | 137.17 | 13.92 |
| GIP-071 | 4.92 | 2.80 | 4.89 | 0.94 | 2.99 | 5.00 | 2.77 | <20 | 24.72 | 459 | male | 40 | 124.32 | 122.42 | 13.67 |
| GIP-072 | 7.16 | 7.40 | 14.00 | 0.77 | 2.82 | 4.40 | 1.72 | <20 | 23.51 | 255 | male | 85 | 75.45 | 128.20 | 19.78 |
| GIP-073 | 7.12 | 4.92 | 3.92 | 1.01 | 3.61 | 5.57 | 2.21 | <20 | 24.57 | 530 | male | 33 | 80.15 | 130.78 | 15.08 |
| GIP-074 | 5.45 | 24.70 | 5.31 | 1.04 | 4.29 | 6.06 | 1.49 | <20 | 26.15 | 480 | male | 64 | 118.69 | 130.75 | 19.02 |
| **CGA group** | | | | | | | | | | | | | | | |
| CGA-001 | 6.16 | 1.70 | 7.56 | 1.07 | 3.14 | 5.50 | 3.14 | <20 | 21.16 | 456 | male | 63 | 251.35 | 127.79 | 27.29 |
| CGA-002 | 5.75 | 13.10 | 10.45 | 1.63 | 3.95 | 6.44 | 1.31 | <20 | 22.85 | 302 | male | 70 | 205.01 | 139.88 | 18.97 |
| CGA-003 | 13.26 | 0.50 | 5.39 | 0.73 | 2.23 | 4.37 | 3.10 | <20 | 21.96 | 259 | male | 69 | 205.99 | 125.22 | 19.82 |
| CGA-004 | 7.86 | 2.00 | 11.14 | 1.69 | 3.06 | 5.41 | 0.71 | <20 | 22.68 | 315 | male | 70 | 246.66 | 143.93 | 22.76 |
| CGA-005 | 5.60 | 10.30 | 12.24 | 0.74 | 2.59 | 5.20 | 4.32 | <20 | 19.65 | 465 | male | 45 | 241.88 | 134.12 | 28.45 |
| CGA-006 | 7.60 | 8.20 | 11.42 | 0.78 | 3.91 | 5.50 | 1.49 | <20 | 19.68 | 219 | male | 88 | 239.67 | 129.76 | 25.80 |
| CGA-007 | 9.01 | 29.50 | 9.29 | 1.77 | 1.64 | 3.97 | 0.66 | <20 | 20.52 | 419 | male | 72 | 205.83 | 122.99 | 27.80 |
| CGA-008 | 6.89 | 0.30 | 6.95 | 1.07 | 3.12 | 4.91 | 1.82 | <20 | 20.09 | 227 | male | 67 | 193.50 | 146.56 | 24.88 |
| CGA-009 | 6.86 | 0.19 | 4.40 | 1.37 | 4.48 | 6.88 | 1.92 | <20 | 21.31 | 319 | male | 65 | 193.49 | 126.95 | 25.08 |
| CGA-010 | 8.41 | 17.85 | 7.46 | 1.74 | 2.23 | 4.42 | 2.17 | <20 | 20.66 | 427 | male | 66 | 231.53 | 123.15 | 31.02 |
| CGA-011 | 9.40 | 12.26 | 9.47 | 1.11 | 2.20 | 4.20 | 0.66 | <20 | 20.15 | 274 | male | 70 | 226.42 | 129.10 | 30.03 |
| CGA-012 | 6.39 | 1.95 | 7.89 | 1.66 | 3.58 | 6.52 | 2.08 | <20 | 21.45 | 329 | male | 66 | 266.04 | 136.92 | 28.88 |
| CGA-013 | 8.67 | 2.26 | 11.54 | 1.71 | 4.02 | 6.09 | 3.00 | <20 | 21.18 | 222 | male | 71 | 226.85 | 128.98 | 26.25 |
| CGA-014 | 9.31 | 13.61 | 11.92 | 1.59 | 3.08 | 5.22 | 1.05 | <20 | 21.61 | 456 | male | 69 | 181.07 | 123.08 | 20.73 |
| CGA-015 | 6.51 | 18.23 | 7.25 | 1.77 | 3.09 | 4.28 | 4.19 | <20 | 22.82 | 247 | male | 69 | 235.24 | 126.95 | 25.80 |
| CGA-016 | 8.08 | 0.86 | 10.86 | 1.02 | 3.11 | 5.35 | 1.09 | <20 | 22.76 | 413 | male | 72 | 210.37 | 145.34 | 17.35 |
| CGA-017 | 7.16 | 3.94 | 8.50 | 0.84 | 3.80 | 4.08 | 0.92 | <20 | 21.54 | 242 | male | 61 | 207.19 | 142.22 | 21.21 |
| CGA-018 | 7.59 | 15.67 | 11.49 | 0.92 | 3.12 | 6.33 | 3.75 | <20 | 21.37 | 234 | male | 66 | 155.10 | 137.85 | 27.58 |
| CGA-019 | 8.55 | 19.54 | 7.24 | 1.76 | 2.37 | 5.31 | 2.93 | <20 | 22.15 | 354 | male | 74 | 176.62 | 126.92 | 19.26 |
| CGA-020 | 6.70 | 6.68 | 8.85 | 1.51 | 2.40 | 6.78 | 1.75 | <20 | 20.87 | 414 | male | 68 | 244.95 | 125.58 | 32.87 |
| CGA-021 | 7.47 | 14.49 | 9.87 | 0.92 | 4.32 | 5.98 | 3.69 | <20 | 20.08 | 288 | male | 64 | 228.82 | 135.35 | 15.85 |
| CGA-022 | 6.87 | 17.87 | 6.38 | 1.72 | 3.22 | 4.08 | 1.29 | <20 | 22.02 | 229 | male | 69 | 221.95 | 135.55 | 15.53 |
| CGA-023 | 8.86 | 19.66 | 8.20 | 1.73 | 1.83 | 4.74 | 2.14 | <20 | 21.53 | 392 | male | 64 | 169.19 | 146.04 | 18.20 |
| CGA-024 | 8.08 | 0.93 | 5.54 | 1.56 | 2.46 | 5.10 | 0.68 | <20 | 22.55 | 433 | male | 66 | 234.97 | 137.53 | 23.37 |
| CGA-025 | 7.34 | 29.12 | 11.89 | 1.32 | 2.62 | 4.39 | 3.88 | <20 | 22.80 | 300 | male | 67 | 155.33 | 146.96 | 27.43 |
| CGA-026 | 9.12 | 27.69 | 8.88 | 1.66 | 2.20 | 4.54 | 2.58 | <20 | 21.72 | 371 | male | 72 | 206.62 | 121.99 | 29.50 |
| CGA-027 | 8.30 | 5.90 | 7.70 | 0.94 | 3.18 | 5.52 | 2.54 | <20 | 21.70 | 323 | male | 67 | 210.20 | 126.57 | 20.70 |
| CGA-028 | 7.04 | 13.95 | 8.11 | 0.96 | 1.89 | 7.00 | 1.93 | <20 | 20.28 | 376 | male | 76 | 140.85 | 149.44 | 21.98 |
| CGA-029 | 6.30 | 12.80 | 5.04 | 1.18 | 1.72 | 6.50 | 2.81 | <20 | 21.49 | 461 | male | 78 | 203.07 | 136.35 | 34.85 |
| CGA-030 | 6.60 | 24.15 | 10.46 | 1.15 | 1.66 | 5.21 | 1.04 | <20 | 19.77 | 230 | male | 83 | 265.35 | 134.79 | 17.34 |
| CGA-031 | 6.62 | 8.58 | 8.69 | 1.33 | 4.47 | 5.67 | 0.75 | <20 | 21.89 | 399 | male | 66 | 169.26 | 128.55 | 24.39 |
| CGA-032 | 7.33 | 9.24 | 7.97 | 1.28 | 4.22 | 6.29 | 2.69 | <20 | 20.18 | 444 | male | 74 | 211.27 | 121.31 | 20.79 |
| CGA-033 | 9.17 | 16.91 | 8.16 | 1.07 | 2.22 | 4.00 | 2.00 | <20 | 20.97 | 296 | male | 74 | 249.56 | 123.82 | 30.13 |
| CGA-034 | 6.47 | 13.65 | 8.89 | 1.43 | 2.76 | 5.70 | 1.32 | <20 | 22.54 | 395 | male | 65 | 205.22 | 131.12 | 29.57 |
| CGA-035 | 7.78 | 18.31 | 8.10 | 1.29 | 4.38 | 5.78 | 2.31 | <20 | 21.41 | 252 | male | 60 | 193.92 | 125.16 | 27.03 |
| CGA-036 | 6.73 | 10.39 | 7.52 | 1.57 | 2.78 | 6.23 | 4.30 | <20 | 20.95 | 399 | male | 64 | 263.54 | 122.49 | 26.84 |
| CGA-037 | 6.92 | 14.69 | 7.87 | 1.59 | 2.09 | 4.67 | 1.27 | <20 | 22.38 | 248 | male | 60 | 261.47 | 136.56 | 18.33 |
| CGA-038 | 9.02 | 11.91 | 9.71 | 1.23 | 3.92 | 5.38 | 0.88 | <20 | 21.38 | 321 | male | 73 | 203.59 | 129.07 | 19.12 |
| CGA-039 | 8.11 | 9.11 | 9.91 | 1.24 | 2.27 | 4.51 | 3.64 | <20 | 20.64 | 443 | male | 71 | 231.76 | 146.16 | 23.16 |
| CGA-040 | 9.36 | 11.43 | 10.79 | 0.94 | 2.59 | 5.06 | 3.16 | <20 | 22.41 | 393 | male | 64 | 231.82 | 129.98 | 26.32 |
| CGA-041 | 7.32 | 15.79 | 8.53 | 1.17 | 3.37 | 6.24 | 4.11 | <20 | 20.04 | 253 | female | 65 | 226.84 | 141.28 | 26.98 |
| CGA-042 | 8.39 | 1.48 | 5.06 | 1.76 | 3.60 | 6.59 | 0.74 | <20 | 22.79 | 341 | male | 71 | 188.29 | 125.74 | 19.04 |
| CGA-043 | 7.27 | 6.19 | 8.73 | 1.63 | 3.87 | 5.38 | 1.19 | <20 | 21.12 | 238 | male | 83 | 230.87 | 130.49 | 26.14 |
| CGA-044 | 9.14 | 8.10 | 9.03 | 0.93 | 4.47 | 4.38 | 4.09 | <20 | 21.59 | 313 | male | 63 | 190.54 | 140.07 | 17.43 |
| CGA-045 | 7.63 | 11.58 | 7.75 | 1.17 | 3.89 | 4.94 | 3.14 | <20 | 21.35 | 293 | male | 68 | 205.24 | 137.08 | 28.42 |
| CGA-046 | 7.90 | 15.66 | 8.09 | 1.52 | 2.05 | 6.73 | 2.68 | <20 | 22.80 | 267 | male | 75 | 254.22 | 122.22 | 25.98 |
| CGA-047 | 7.72 | 17.80 | 11.31 | 1.26 | 2.10 | 5.44 | 1.18 | <20 | 21.53 | 448 | male | 75 | 210.67 | 138.11 | 17.97 |
| CGA-048 | 6.79 | 5.38 | 7.67 | 1.61 | 2.17 | 5.69 | 0.75 | <20 | 20.83 | 255 | male | 74 | 213.79 | 140.56 | 27.14 |
| CGA-049 | 6.67 | 9.34 | 10.10 | 1.67 | 2.01 | 5.73 | 0.81 | <20 | 20.73 | 301 | male | 69 | 245.99 | 131.56 | 24.11 |
| CGA-050 | 7.87 | 10.96 | 5.51 | 1.67 | 1.68 | 4.87 | 1.29 | <20 | 20.64 | 297 | male | 64 | 205.64 | 131.14 | 20.08 |
| CGA-051 | 7.32 | 12.41 | 7.95 | 1.60 | 3.31 | 5.26 | 2.79 | <20 | 21.35 | 453 | male | 62 | 147.55 | 133.62 | 29.82 |
| CGA-052 | 8.52 | 7.58 | 9.79 | 1.05 | 4.06 | 5.48 | 1.25 | <20 | 22.57 | 267 | male | 80 | 181.74 | 125.40 | 27.74 |
| CGA-053 | 8.77 | 10.30 | 10.39 | 1.55 | 1.76 | 6.10 | 2.96 | <20 | 22.76 | 419 | male | 74 | 169.20 | 143.39 | 30.50 |
| CGA-054 | 7.08 | 9.07 | 11.19 | 1.23 | 3.46 | 4.38 | 2.93 | <20 | 20.41 | 435 | male | 82 | 231.84 | 140.32 | 19.15 |
| CGA-055 | 8.77 | 15.17 | 7.10 | 1.02 | 4.03 | 5.65 | 1.45 | <20 | 20.02 | 407 | male | 72 | 200.96 | 140.27 | 24.58 |
| CGA-056 | 9.12 | 12.74 | 7.30 | 1.18 | 3.04 | 4.27 | 1.89 | <20 | 21.43 | 344 | male | 67 | 201.06 | 138.76 | 19.54 |
| CGA-057 | 7.65 | 5.17 | 9.88 | 0.98 | 4.27 | 4.69 | 0.85 | <20 | 19.88 | 341 | female | 65 | 161.42 | 133.31 | 21.76 |
| CGA-058 | 6.79 | 12.02 | 8.35 | 1.01 | 3.20 | 5.40 | 1.40 | <20 | 20.07 | 272 | male | 64 | 170.72 | 128.84 | 26.11 |
| CGA-059 | 6.92 | 9.81 | 7.15 | 1.39 | 3.03 | 6.28 | 1.40 | <20 | 20.43 | 322 | male | 64 | 194.83 | 130.13 | 26.74 |
| CGA-060 | 6.20 | 15.98 | 9.41 | 1.24 | 1.97 | 6.72 | 2.46 | <20 | 20.80 | 383 | male | 65 | 254.16 | 123.17 | 18.20 |
| CGA-061 | 7.74 | 10.43 | 10.65 | 1.62 | 3.14 | 5.16 | 3.57 | <20 | 22.65 | 371 | male | 75 | 188.32 | 139.35 | 21.19 |
| CGA-062 | 6.07 | 13.36 | 8.09 | 1.56 | 2.03 | 5.41 | 2.11 | <20 | 21.40 | 390 | male | 60 | 224.27 | 137.27 | 24.35 |
| **RA group** | | | | | | | | | | | | | | | |
| RA-001 | 8.86 | 31.10 | 4.52 | 1.34 | 2.45 | 4.12 | 0.96 | 970 | 24.54 | 217 | female | 65 | 137.21 | 135.34 | 21.52 |
| RA-002 | 13.19 | 56.00 | 12.86 | 1.25 | 2.81 | 4.79 | 1.31 | 568 | 22.00 | 483 | female | 72 | 95.30 | 122.39 | 28.05 |
| RA-003 | 7.04 | 41.20 | 5.09 | 1.08 | 2.72 | 4.51 | 1.42 | 808 | 24.58 | 306 | female | 59 | 119.64 | 150.00 | 20.35 |
| RA-004 | 7.47 | 4.30 | 4.87 | 1.96 | 4.01 | 6.67 | 1.07 | 19.9 | 23.92 | 200 | female | 71 | 122.12 | 131.65 | 30.01 |
| RA-005 | 9.57 | 75.60 | 3.25 | 1.06 | 2.13 | 3.92 | 1.82 | 513 | 21.39 | 203 | female | 58 | 105.54 | 140.92 | 27.95 |
| RA-006 | 11.73 | 10.90 | 4.38 | 1.06 | 2.69 | 4.19 | 0.84 | 160 | 19.77 | 255 | female | 47 | 70.43 | 125.93 | 18.79 |
| RA-007 | 5.51 | 7.60 | 3.63 | 1.16 | 1.87 | 3.59 | 1.61 | 19.9 | 19.85 | 230 | female | 56 | 119.90 | 140.42 | 26.62 |
| RA-008 | 8.21 | 13.70 | 7.51 | 2.18 | 4.30 | 7.20 | 0.75 | 730 | 21.44 | 288 | female | 73 | 135.34 | 129.57 | 30.13 |
| RA-009 | 7.77 | 2.70 | 5.80 | 1.34 | 3.53 | 5.49 | 0.83 | 19.9 | 19.65 | 185 | male | 45 | 136.53 | 143.05 | 25.20 |
| RA-010 | 6.14 | 2.40 | 5.57 | 2.14 | 3.03 | 4.59 | 1.63 | 19.9 | 19.20 | 276 | female | 28 | 135.83 | 141.78 | 32.37 |
| RA-011 | 7.01 | 104.00 | 4.34 | 1.58 | 1.26 | 3.31 | 0.92 | 19.9 | 23.14 | 317 | female | 61 | 66.52 | 145.00 | 25.55 |
| RA-012 | 10.22 | 21.60 | 4.12 | 0.88 | 2.75 | 4.08 | 1.27 | 19.9 | 23.78 | 414 | female | 39 | 132.89 | 141.12 | 33.46 |
| RA-013 | 5.04 | 1.50 | 4.26 | 1.85 | 2.54 | 4.39 | 0.68 | 45 | 20.90 | 138 | female | 42 | 72.47 | 142.80 | 22.51 |
| RA-014 | 4.49 | 1.10 | 4.56 | 1.54 | 3.18 | 5.26 | 0.70 | 19.9 | 24.02 | 230 | female | 55 | 107.83 | 125.48 | 26.48 |
| RA-015 | 10.59 | 6.10 | 5.28 | 1.84 | 2.93 | 4.23 | 1.86 | 19.9 | 22.95 | 276 | male | 50 | 124.29 | 145.32 | 26.90 |
| RA-016 | 6.33 | 10.60 | 4.61 | 0.83 | 2.60 | 4.08 | 1.90 | 19.9 | 19.80 | 388 | male | 57 | 75.42 | 136.13 | 15.33 |
| RA-017 | 6.07 | 5.90 | 8.00 | 1.38 | 3.59 | 5.60 | 1.41 | 878 | 18.69 | 251 | female | 68 | 109.57 | 134.95 | 19.61 |
| RA-018 | 3.54 | 2.00 | 8.90 | 1.68 | 3.36 | 5.58 | 0.80 | 711 | 22.67 | 344 | female | 66 | 81.29 | 128.74 | 19.84 |
| RA-019 | 5.00 | 18.20 | 4.01 | 1.21 | 2.12 | 3.74 | 0.63 | 529 | 19.87 | 179 | female | 64 | 105.08 | 136.77 | 27.82 |
| RA-020 | 4.50 | 20.00 | 4.92 | 0.91 | 2.37 | 4.16 | 1.41 | 19.9 | 20.93 | 138 | female | 53 | 109.37 | 120.35 | 21.42 |
| RA-021 | 6.80 | 23.20 | 5.00 | 1.54 | 3.28 | 4.31 | 0.72 | 20.4 | 19.17 | 344 | female | 51 | 68.75 | 125.68 | 32.17 |
| RA-022 | 6.15 | 10.30 | 6.62 | 1.22 | 1.65 | 3.45 | 0.95 | 379 | 23.48 | 272 | female | 67 | 98.89 | 139.11 | 17.64 |
| RA-023 | 15.22 | 34.50 | 10.23 | 1.94 | 1.55 | 4.42 | 1.00 | 656 | 22.42 | 288 | male | 71 | 102.84 | 137.26 | 18.48 |
| RA-024 | 5.44 | 15.30 | 10.13 | 1.44 | 1.18 | 3.02 | 0.63 | 67.9 | 21.77 | 451 | male | 52 | 89.21 | 125.79 | 25.19 |
| RA-025 | 7.02 | 15.97 | 5.55 | 1.92 | 3.93 | 7.15 | 1.49 | 495.81 | 21.43 | 242 | female | 54 | 91.61 | 149.73 | 23.82 |
| RA-026 | 5.86 | 31.95 | 5.60 | 1.56 | 3.35 | 6.09 | 1.44 | 250.06 | 23.16 | 396 | female | 46 | 119.96 | 148.40 | 19.03 |
| RA-027 | 8.93 | 15.13 | 4.31 | 2.05 | 3.07 | 5.08 | 0.90 | 272.58 | 20.95 | 285 | female | 58 | 127.61 | 137.75 | 17.00 |
| RA-028 | 9.57 | 23.02 | 3.72 | 1.47 | 2.43 | 5.67 | 1.59 | 381.3 | 24.32 | 468 | female | 51 | 108.88 | 121.72 | 30.65 |
| RA-029 | 5.74 | 29.41 | 4.55 | 1.33 | 3.93 | 5.96 | 1.20 | 169.87 | 23.68 | 328 | female | 52 | 75.21 | 134.65 | 30.29 |
| RA-030 | 7.83 | 27.55 | 3.87 | 1.82 | 3.71 | 3.34 | 0.96 | 105.71 | 20.56 | 438 | female | 47 | 94.54 | 125.02 | 22.92 |
| RA-031 | 7.01 | 33.73 | 7.18 | 1.28 | 2.69 | 3.92 | 1.69 | 144.76 | 18.76 | 221 | male | 73 | 97.53 | 138.80 | 26.60 |
| RA-032 | 6.28 | 5.69 | 8.47 | 1.97 | 2.77 | 5.55 | 0.96 | 228.73 | 20.83 | 264 | female | 49 | 96.43 | 131.72 | 34.30 |
