# Supplementary material for: Establishment of a clinical diagnostic model for gouty arthritis based on the serum biochemical profile: A case-control study
Source: Medicine (Baltimore). 2021 Apr 23;100(16):e25542. doi: 10.1097/MD.0000000000025542 (PMC8078334; doi:10.1097/MD.0000000000025542)
Supplement: Supplemental Digital Content [file medi-100-e25542-s005.doc]

**Table S2. Raw data of biochemical indicators in serum specimens of validation set（n=200）**

| **NO.** | **WBC**  **(10∧9/L)** | **CRP**  **(Mg/L)** | **BUN**  **(mmol/L)** | **HDL**  **(mmol/L)** | **LDL**  **(mmol/L)** | **TC**  **(mmol/L)** | **TG**  **(mmol/L)** | **RF** | **BMI**  **(kg/m^2^)** | **UA**  **(μmol/L)** | **Sex** | **Age** | **Creatinine**  **(μmol/L)** | **Hemoglobin**  **(g/L)** | **ESR**  **(mm/h)** |
| --- | --- | --- | --- | --- | --- | --- | --- | --- | --- | --- | --- | --- | --- | --- | --- |
| **Control group** | | | | | | | | | | | | | | | |
| Control-081 | 5.55 | 0.50 | 5.90 | 0.98 | 2.14 | 5.25 | 3.67 | <20 | 26.88 | 170 | female | 64 | 60.13 | 132.19 | 10.44 |
| Control-082 | 6.69 | 0.50 | 5.22 | 1.27 | 3.66 | 5.77 | 1.43 | <20 | 22.12 | 221 | male | 47 | 91.79 | 140.83 | 16.31 |
| Control-083 | 8.83 | 2.00 | 6.95 | 0.96 | 2.69 | 4.73 | 2.08 | <20 | 20.45 | 205 | male | 54 | 96.02 | 122.80 | 20.00 |
| Control-084 | 5.27 | 0.50 | 5.26 | 1.04 | 3.05 | 5.25 | 2.04 | <20 | 26.74 | 196 | male | 47 | 72.39 | 126.49 | 15.96 |
| Control-085 | 5.48 | 0.50 | 4.49 | 1.15 | 3.72 | 5.37 | 1.01 | <20 | 24.71 | 195 | male | 59 | 98.18 | 134.94 | 13.63 |
| Control-086 | 3.71 | 0.50 | 6.87 | 1.35 | 2.75 | 4.77 | 0.94 | <20 | 23.76 | 194 | male | 64 | 87.03 | 133.73 | 10.84 |
| Control-087 | 6.64 | 0.50 | 4.46 | 1.63 | 3.12 | 5.51 | 0.82 | <20 | 27.80 | 191 | female | 49 | 70.90 | 126.55 | 18.38 |
| Control-088 | 5.38 | 0.50 | 3.75 | 1.55 | 3.97 | 6.21 | 1.13 | <20 | 24.65 | 197 | female | 63 | 91.35 | 138.35 | 17.89 |
| Control-089 | 5.94 | 0.50 | 5.29 | 1.01 | 3.06 | 5.26 | 2.25 | <20 | 23.09 | 203 | male | 42 | 83.49 | 126.83 | 14.91 |
| Control-090 | 5.82 | 0.30 | 5.30 | 2.09 | 3.55 | 6.47 | 1.74 | <20 | 19.73 | 225 | female | 63 | 55.73 | 134.27 | 18.30 |
| Control-091 | 5.96 | 0.60 | 6.52 | 1.08 | 3.66 | 5.54 | 1.22 | <20 | 25.37 | 217 | male | 42 | 87.21 | 120.53 | 10.49 |
| Control-092 | 4.44 | 0.50 | 4.86 | 1.67 | 3.22 | 5.74 | 0.79 | <20 | 18.83 | 55 | male | 54 | 91.08 | 149.61 | 19.06 |
| Control-093 | 5.40 | 0.40 | 3.70 | 1.59 | 2.46 | 4.87 | 1.87 | <20 | 20.57 | 67 | female | 53 | 89.95 | 141.60 | 18.21 |
| Control-094 | 5.04 | 0.30 | 3.81 | 1.58 | 1.82 | 3.91 | 0.56 | <20 | 21.76 | 50 | female | 43 | 59.14 | 136.12 | 12.89 |
| Control-095 | 6.41 | 0.60 | 4.53 | 0.99 | 1.89 | 3.35 | 0.73 | <20 | 26.68 | 59 | male | 56 | 65.07 | 142.47 | 17.89 |
| Control-096 | 3.95 | 0.20 | 4.95 | 1.50 | 4.01 | 6.30 | 0.87 | <20 | 20.42 | 65 | male | 64 | 71.91 | 139.84 | 14.16 |
| Control-097 | 5.69 | 0.20 | 6.18 | 1.10 | 2.71 | 4.25 | 0.59 | <20 | 24.17 | 68 | male | 51 | 59.39 | 142.22 | 15.50 |
| Control-098 | 4.92 | 0.30 | 4.19 | 2.01 | 3.04 | 5.89 | 1.18 | <20 | 27.09 | 40 | female | 57 | 68.21 | 136.95 | 13.68 |
| Control-099 | 6.92 | 0.50 | 4.05 | 1.35 | 4.16 | 6.52 | 1.27 | <20 | 20.42 | 50 | female | 55 | 92.12 | 142.31 | 18.11 |
| Control-100 | 10.63 | 3.80 | 5.58 | 1.30 | 3.77 | 5.80 | 1.23 | <20 | 22.24 | 202 | male | 47 | 83.73 | 134.58 | 17.06 |
| Control-101 | 5.10 | 0.40 | 3.93 | 1.64 | 2.35 | 4.30 | 0.55 | <20 | 23.74 | 179 | female | 44 | 76.29 | 120.29 | 15.55 |
| Control-102 | 6.37 | 0.40 | 5.76 | 0.71 | 3.52 | 5.11 | 1.53 | <20 | 22.07 | 70 | male | 63 | 64.36 | 140.11 | 15.69 |
| Control-103 | 5.63 | 0.40 | 3.63 | 1.26 | 3.38 | 5.67 | 1.48 | <20 | 21.26 | 81 | male | 47 | 91.58 | 145.89 | 12.46 |
| Control-104 | 5.58 | 0.40 | 5.09 | 2.10 | 3.23 | 6.17 | 0.83 | <20 | 22.70 | 127 | female | 55 | 60.33 | 129.35 | 14.45 |
| Control-105 | 6.32 | 0.40 | 5.39 | 1.00 | 2.97 | 5.03 | 2.91 | <20 | 25.68 | 201 | male | 52 | 53.48 | 137.42 | 15.48 |
| Control-106 | 3.12 | 0.60 | 4.08 | 1.46 | 3.30 | 5.50 | 0.69 | <20 | 22.18 | 205 | female | 53 | 57.14 | 143.04 | 18.03 |
| Control-107 | 5.26 | 0.70 | 3.36 | 1.31 | 2.89 | 5.08 | 1.38 | <20 | 27.33 | 73 | female | 52 | 86.47 | 131.71 | 19.21 |
| Control-108 | 4.70 | 0.40 | 3.84 | 1.64 | 3.44 | 5.81 | 1.27 | <20 | 19.93 | 120 | female | 46 | 86.74 | 134.40 | 10.17 |
| Control-109 | 5.71 | 0.30 | 6.29 | 1.31 | 3.45 | 5.58 | 1.27 | <20 | 19.12 | 112 | male | 59 | 50.29 | 125.66 | 19.35 |
| Control-110 | 3.59 | 0.30 | 4.50 | 2.21 | 2.63 | 5.52 | 0.88 | <20 | 25.13 | 2 | female | 45 | 52.73 | 149.97 | 15.65 |
| Control-111 | 7.09 | 1.00 | 4.23 | 1.34 | 2.29 | 4.45 | 2.60 | <20 | 22.30 | 131 | male | 42 | 56.33 | 126.42 | 17.01 |
| Control-112 | 4.41 | 0.30 | 4.95 | 1.90 | 3.58 | 5.50 | 0.76 | <20 | 24.34 | 137 | female | 42 | 60.01 | 124.05 | 16.23 |
| Control-113 | 8.37 | 2.20 | 5.24 | 0.80 | 3.58 | 5.36 | 1.76 | <20 | 22.96 | 138 | male | 55 | 72.46 | 138.74 | 13.71 |
| Control-114 | 4.48 | 0.30 | 3.98 | 1.26 | 2.43 | 4.54 | 2.32 | <20 | 21.86 | 132 | female | 45 | 88.70 | 141.71 | 14.38 |
| Control-115 | 4.71 | 0.30 | 5.18 | 1.12 | 3.98 | 6.00 | 1.27 | <20 | 27.85 | 28 | male | 55 | 85.33 | 140.98 | 11.84 |
| Control-116 | 4.81 | 0.30 | 5.15 | 1.83 | 2.00 | 4.50 | 0.43 | <20 | 20.13 | 338 | female | 47 | 54.42 | 138.98 | 18.00 |
| Control-117 | 8.53 | 2.40 | 5.50 | 1.29 | 2.70 | 4.48 | 0.84 | <20 | 20.81 | 182 | male | 58 | 89.59 | 122.22 | 14.70 |
| Control-118 | 6.30 | 0.30 | 5.21 | 1.37 | 2.41 | 4.42 | 0.72 | <20 | 18.73 | 334 | female | 43 | 73.59 | 131.72 | 14.44 |
| Control-119 | 4.98 | 0.30 | 4.04 | 1.90 | 3.53 | 6.35 | 0.87 | <20 | 25.25 | 336 | male | 53 | 69.02 | 137.16 | 17.18 |
| Control-120 | 4.42 | 0.30 | 6.00 | 1.33 | 1.40 | 3.12 | 0.62 | <20 | 22.36 | 194 | female | 43 | 93.60 | 132.51 | 11.23 |
| Control-121 | 8.27 | 0.30 | 4.21 | 1.21 | 4.17 | 6.71 | 2.11 | <20 | 23.79 | 198 | male | 61 | 89.31 | 136.67 | 14.81 |
| Control-122 | 3.99 | 0.30 | 4.97 | 1.40 | 3.39 | 5.49 | 0.86 | <20 | 19.34 | 294 | female | 45 | 69.90 | 121.47 | 13.36 |
| Control-123 | 7.72 | 1.10 | 6.87 | 1.40 | 3.14 | 5.38 | 1.03 | <20 | 19.47 | 229 | male | 56 | 78.97 | 140.14 | 17.76 |
| Control-124 | 4.66 | 0.30 | 6.15 | 2.00 | 2.85 | 5.48 | 0.72 | <20 | 20.70 | 67 | female | 43 | 92.96 | 136.72 | 10.94 |
| Control-125 | 3.82 | 0.30 | 4.63 | 1.23 | 2.42 | 4.20 | 1.08 | <20 | 21.69 | 86 | female | 49 | 87.35 | 147.89 | 16.31 |
| Control-126 | 6.06 | 0.30 | 3.65 | 1.69 | 3.44 | 5.92 | 0.95 | <20 | 19.24 | 263 | female | 47 | 82.03 | 130.22 | 12.38 |
| Control-127 | 6.38 | 0.30 | 4.42 | 1.05 | 3.15 | 5.19 | 1.47 | <20 | 20.15 | 226 | female | 60 | 83.57 | 138.10 | 12.81 |
| Control-128 | 6.25 | 0.30 | 6.18 | 1.03 | 3.53 | 5.89 | 2.48 | <20 | 22.78 | 259 | male | 63 | 51.20 | 123.44 | 14.49 |
| Control-129 | 5.03 | 0.30 | 6.12 | 2.03 | 3.06 | 5.95 | 0.71 | <20 | 23.26 | 272 | female | 44 | 77.94 | 146.66 | 11.76 |
| Control-130 | 6.91 | 0.30 | 5.03 | 1.79 | 2.57 | 5.19 | 1.08 | <20 | 26.51 | 260 | female | 45 | 57.79 | 131.30 | 19.57 |
| Control-131 | 6.95 | 0.30 | 5.62 | 1.51 | 2.92 | 5.17 | 0.89 | <20 | 25.48 | 204 | male | 60 | 55.16 | 137.60 | 14.55 |
| Control-132 | 7.55 | 1.20 | 3.05 | 1.87 | 2.40 | 4.85 | 1.59 | <20 | 26.58 | 267 | female | 42 | 99.22 | 128.58 | 19.03 |
| Control-133 | 4.02 | 0.30 | 3.85 | 1.31 | 2.53 | 4.29 | 1.11 | <20 | 18.55 | 115 | male | 49 | 70.59 | 134.31 | 12.01 |
| Control-134 | 4.13 | 0.30 | 4.37 | 1.31 | 1.63 | 3.54 | 1.19 | <20 | 22.29 | 236 | male | 48 | 51.17 | 145.69 | 14.42 |
| Control-135 | 5.67 | 0.30 | 4.16 | 1.66 | 2.57 | 4.94 | 0.91 | <20 | 27.47 | 122 | male | 55 | 77.35 | 135.00 | 12.24 |
| Control-136 | 5.54 | 0.30 | 5.64 | 1.79 | 1.81 | 4.38 | 0.68 | <20 | 23.35 | 294 | male | 62 | 63.46 | 120.87 | 13.13 |
| Control-137 | 6.36 | 0.30 | 3.79 | 1.60 | 2.05 | 4.08 | 0.84 | <20 | 24.71 | 92 | female | 41 | 91.32 | 121.96 | 12.16 |
| Control-138 | 8.58 | 0.30 | 5.89 | 1.09 | 2.70 | 4.69 | 1.57 | <20 | 27.69 | 279 | female | 67 | 83.97 | 142.43 | 15.69 |
| Control-139 | 7.71 | 1.20 | 6.16 | 1.19 | 3.26 | 5.45 | 1.03 | <20 | 22.74 | 83 | male | 72 | 60.88 | 148.88 | 10.39 |
| Control-140 | 6.28 | 0.30 | 5.70 | 0.99 | 1.68 | 3.28 | 1.00 | <20 | 27.35 | 79 | male | 71 | 83.47 | 136.98 | 17.86 |
| Control-141 | 5.88 | 0.30 | 5.20 | 1.58 | 2.40 | 4.68 | 0.76 | <20 | 19.10 | 182 | female | 58 | 66.22 | 135.97 | 13.28 |
| Control-142 | 4.51 | 0.30 | 5.77 | 1.08 | 3.18 | 5.35 | 1.48 | <20 | 25.42 | 172 | female | 61 | 59.64 | 140.26 | 17.53 |
| Control-143 | 8.07 | 2.30 | 6.14 | 1.15 | 2.38 | 4.41 | 1.89 | <20 | 27.30 | 268 | male | 73 | 55.03 | 128.67 | 18.83 |
| Control-144 | 10.66 | 2.50 | 6.40 | 1.13 | 1.82 | 3.50 | 1.43 | <20 | 25.54 | 266 | male | 60 | 96.93 | 147.52 | 15.46 |
| Control-145 | 3.96 | 0.30 | 7.07 | 1.16 | 3.88 | 6.17 | 1.53 | <20 | 24.62 | 258 | female | 61 | 64.63 | 138.42 | 14.05 |
| Control-146 | 6.49 | 0.30 | 7.68 | 1.22 | 2.41 | 4.31 | 0.96 | <20 | 19.19 | 73 | male | 57 | 82.49 | 124.59 | 19.25 |
| Control-147 | 5.12 | 0.30 | 5.73 | 0.74 | 1.52 | 3.22 | 2.32 | <20 | 21.26 | 74 | female | 49 | 91.92 | 139.73 | 16.02 |
| Control-148 | 4.63 | 0.30 | 3.29 | 1.70 | 2.21 | 4.85 | 1.64 | <20 | 26.57 | 57 | female | 41 | 53.00 | 133.81 | 11.95 |
| Control-149 | 7.04 | 0.30 | 3.25 | 1.12 | 1.99 | 3.81 | 1.43 | <20 | 25.57 | 267 | male | 55 | 93.70 | 127.44 | 14.00 |
| Control-150 | 9.97 | 2.50 | 3.92 | 0.96 | 1.91 | 3.59 | 1.73 | <20 | 24.51 | 324 | male | 50 | 54.54 | 136.08 | 10.76 |
| Control-151 | 12.98 | 5.90 | 4.50 | 1.06 | 3.82 | 5.58 | 0.70 | <20 | 22.79 | 11 | male | 56 | 53.89 | 121.92 | 15.52 |
| Control-152 | 5.83 | 1.80 | 3.33 | 1.11 | 2.68 | 4.60 | 1.04 | <20 | 23.67 | 226 | female | 58 | 83.48 | 145.94 | 12.73 |
| Control-153 | 3.09 | 0.30 | 3.74 | 1.16 | 2.24 | 4.36 | 1.35 | <20 | 26.63 | 94 | female | 71 | 56.33 | 126.88 | 17.57 |
| Control-154 | 5.05 | 0.30 | 3.08 | 1.39 | 1.98 | 3.77 | 0.65 | <20 | 20.09 | 97 | female | 41 | 91.47 | 131.05 | 19.54 |
| Control-155 | 7.85 | 1.60 | 4.91 | 2.12 | 2.44 | 5.28 | 0.67 | <20 | 25.99 | 75 | male | 71 | 88.36 | 130.99 | 17.48 |
| Control-156 | 3.99 | 0.30 | 3.90 | 1.38 | 2.51 | 4.96 | 1.59 | <20 | 21.17 | 338 | female | 55 | 63.90 | 149.64 | 19.90 |
| Control-157 | 5.04 | 0.30 | 5.99 | 0.85 | 3.08 | 4.59 | 1.05 | <20 | 25.75 | 274 | male | 60 | 78.05 | 136.50 | 15.56 |
| Control-158 | 4.38 | 0.30 | 4.67 | 1.01 | 2.39 | 4.34 | 2.07 | <20 | 20.02 | 113 | female | 48 | 61.06 | 146.19 | 19.59 |
| Control-159 | 7.82 | 1.60 | 3.77 | 1.69 | 1.83 | 4.31 | 1.31 | <20 | 21.67 | 226 | female | 59 | 95.53 | 124.02 | 11.39 |
| Control-160 | 4.96 | 0.30 | 4.49 | 1.40 | 3.60 | 6.07 | 1.87 | <20 | 18.59 | 181 | male | 64 | 53.98 | 143.53 | 10.21 |
| **HUA group** | | | | | | | | | | | | | | | |
| HUA-063 | 5.11 | 0.67 | 2.82 | 1.29 | 2.15 | 4.23 | 4.34 | <20 | 26.36 | 509 | male | 68 | 95.13 | 133.41 | 15.8 |
| HUA-064 | 4.62 | 1.46 | 2.14 | 1.86 | 3.43 | 3.11 | 2.26 | <20 | 25.07 | 556 | male | 20 | 58.15 | 129.92 | 20.2 |
| HUA-065 | 6.12 | 0.94 | 3.56 | 1.38 | 3.06 | 3.37 | 3.77 | <20 | 26.49 | 431 | male | 37 | 91.42 | 125.55 | 20.39 |
| HUA-066 | 6.01 | 1.55 | 3.52 | 2.19 | 1.89 | 5.36 | 1.52 | <20 | 24.06 | 470 | male | 51 | 105.3 | 135.1 | 17.35 |
| HUA-067 | 5.38 | 1.43 | 4.12 | 1.45 | 3.13 | 5.52 | 1.48 | <20 | 25.01 | 395 | female | 52 | 90.29 | 139.3 | 20.06 |
| HUA-068 | 7.02 | 0.9 | 4.25 | 0.89 | 2.89 | 5.02 | 2.62 | <20 | 27.16 | 393 | female | 67 | 100.59 | 139.52 | 17.11 |
| HUA-069 | 5.38 | 1.24 | 3.7 | 2.48 | 2.9 | 6.11 | 3.92 | <20 | 26.08 | 471 | male | 44 | 62.14 | 122.2 | 16.74 |
| HUA-070 | 7.43 | 0.86 | 5.72 | 2.15 | 3.68 | 5.83 | 1.37 | <20 | 26.39 | 428 | female | 69 | 72.88 | 133.31 | 20.41 |
| HUA-071 | 4.23 | 0.87 | 5.29 | 1.3 | 3.48 | 4.87 | 3.16 | <20 | 26.52 | 420 | male | 34 | 86.24 | 126.09 | 20.27 |
| HUA-072 | 3.91 | 1.33 | 6.34 | 1.27 | 1.74 | 3.6 | 0.79 | <20 | 26.93 | 511 | male | 15 | 82.88 | 124.22 | 20.8 |
| HUA-073 | 5.52 | 0.89 | 5.92 | 1.76 | 1.76 | 4.26 | 1.05 | <20 | 27.44 | 512 | male | 28 | 68.81 | 142.15 | 18.72 |
| HUA-074 | 5.22 | 1.59 | 4.87 | 0.94 | 2.82 | 4.23 | 0.91 | <20 | 24.49 | 454 | male | 35 | 56.06 | 125.11 | 16.6 |
| HUA-075 | 6.27 | 0.84 | 4.99 | 0.99 | 2.71 | 4.64 | 2.11 | <20 | 25.79 | 360 | female | 65 | 87.45 | 140.99 | 19.23 |
| HUA-076 | 4.3 | 1.1 | 5.27 | 1.82 | 1.75 | 3.32 | 3.15 | <20 | 25.28 | 505 | male | 25 | 62.24 | 128.46 | 20.94 |
| HUA-077 | 5.54 | 0.61 | 4.52 | 1.02 | 2.95 | 5.49 | 4.69 | <20 | 25.12 | 557 | male | 37 | 59.51 | 127.54 | 18.32 |
| HUA-078 | 6.55 | 0.8 | 2 | 1.52 | 1.9 | 3.88 | 2.76 | <20 | 22.6 | 583 | male | 74 | 94.48 | 138.07 | 19.93 |
| HUA-079 | 4.53 | 0.91 | 6.34 | 2.51 | 1.9 | 3.41 | 2.51 | <20 | 24.26 | 493 | female | 21 | 99.49 | 134.58 | 20.37 |
| HUA-080 | 4.44 | 1.21 | 2.88 | 1.31 | 2.75 | 4.83 | 1.74 | <20 | 27.31 | 508 | male | 71 | 88.74 | 143.8 | 19.51 |
| HUA-081 | 4.86 | 1.26 | 5.6 | 2.55 | 2.45 | 4.34 | 3.08 | <20 | 21.11 | 414 | male | 70 | 54.51 | 121.17 | 19.79 |
| HUA-082 | 4.69 | 0.69 | 3.52 | 0.93 | 3.09 | 3.98 | 1.15 | <20 | 26.71 | 401 | male | 59 | 80.66 | 149.52 | 20.89 |
| HUA-083 | 6.86 | 1.48 | 5.46 | 2.01 | 2.4 | 5.35 | 1.8 | <20 | 25.41 | 387 | male | 51 | 96 | 147.78 | 16.49 |
| HUA-084 | 4.49 | 0.77 | 6.86 | 1.56 | 3.17 | 3.2 | 1.19 | <20 | 18.72 | 508 | male | 61 | 51.12 | 147.59 | 19.12 |
| HUA-085 | 4.54 | 1.45 | 5.26 | 1.9 | 2.64 | 3.74 | 3.48 | <20 | 26.68 | 403 | male | 27 | 69.95 | 144.46 | 17.51 |
| HUA-086 | 5.92 | 1.26 | 3 | 1.99 | 2.62 | 4.15 | 2.55 | <20 | 24.49 | 489 | male | 52 | 67.92 | 149.5 | 16.44 |
| HUA-087 | 6.02 | 1.43 | 4.43 | 1.59 | 2.82 | 5.01 | 1.04 | <20 | 26.65 | 481 | male | 28 | 103.22 | 133.15 | 17.52 |
| HUA-088 | 4.93 | 1.11 | 5.32 | 1.63 | 2.45 | 4.26 | 3.86 | <20 | 25 | 442 | male | 69 | 74.19 | 137.65 | 18.88 |
| HUA-089 | 6.06 | 1.6 | 6.77 | 2.11 | 1.51 | 4.53 | 2.61 | <20 | 26.25 | 500 | male | 58 | 66.83 | 132.87 | 15.92 |
| HUA-090 | 4.81 | 0.91 | 3.6 | 2.24 | 2.25 | 5.51 | 4.21 | <20 | 24.79 | 530 | female | 46 | 85.55 | 129.16 | 20.62 |
| HUA-091 | 5.72 | 1.02 | 5.53 | 1.27 | 2.64 | 6.32 | 1.66 | <20 | 21.84 | 384 | male | 32 | 57.72 | 137.52 | 14.21 |
| HUA-092 | 6.32 | 0.81 | 6.13 | 2.47 | 2.26 | 5.16 | 1.03 | <20 | 25.27 | 365 | male | 58 | 92.56 | 122.54 | 17.89 |
| **AGA group** | | | | | | | | | | | | | | | |
| AGA-070 | 7.76 | 32.8 | 7.59 | 0.95 | 4.1 | 5.76 | 2.51 | <20 | 26.49 | 486 | male | 43 | 73.42 | 135.97 | 39.72 |
| AGA-071 | 11.64 | 55.46 | 3.03 | 0.93 | 3.08 | 5.83 | 4.31 | <20 | 29.72 | 570 | male | 42 | 71.05 | 125.2 | 27.58 |
| AGA-072 | 10.12 | 58.25 | 2.82 | 1.04 | 3.96 | 5.78 | 2.49 | <20 | 25.2 | 623 | male | 35 | 76.4 | 132.67 | 37.91 |
| AGA-073 | 10.79 | 56.36 | 2.54 | 0.77 | 2.89 | 4.31 | 2.02 | <20 | 24.02 | 602 | male | 61 | 100.87 | 144.05 | 41.63 |
| AGA-074 | 7.89 | 38.25 | 4.44 | 1.03 | 3.22 | 4.96 | 2.31 | <20 | 31.66 | 472 | male | 30 | 115.29 | 124.82 | 30.22 |
| AGA-075 | 5.78 | 1.9 | 5.59 | 1.02 | 4.4 | 5.99 | 1.12 | <20 | 29.42 | 359 | female | 54 | 77.34 | 122.19 | 22.86 |
| AGA-076 | 12.61 | 19.4 | 4.65 | 1.13 | 2.13 | 4.16 | 2.41 | <20 | 28.73 | 351 | male | 30 | 128.98 | 132.31 | 31.87 |
| AGA-077 | 6.56 | 8.7 | 5.42 | 0.99 | 3.24 | 5.31 | 2.6 | <20 | 28.19 | 389 | male | 63 | 128.2 | 148.52 | 40.4 |
| AGA-078 | 6.59 | 30.27 | 5.4 | 1.08 | 3.39 | 4.84 | 3.34 | <20 | 29.45 | 268 | male | 66 | 114.22 | 123.21 | 27.8 |
| AGA-079 | 4.06 | 34.6 | 3.23 | 1.29 | 3.18 | 5.75 | 2.38 | <20 | 29.05 | 536 | male | 35 | 88.45 | 125.36 | 38.71 |
| AGA-080 | 9.01 | 30.2 | 3.35 | 1.06 | 3.42 | 5.29 | 1.72 | <20 | 26.75 | 513 | male | 35 | 92.15 | 132.43 | 19.47 |
| AGA-081 | 6.22 | 46.75 | 7.5 | 1.05 | 1.35 | 5.56 | 3.49 | <20 | 28.73 | 493 | male | 47 | 113.75 | 121.98 | 39.79 |
| AGA-082 | 11.95 | 60.03 | 2.45 | 0.82 | 2.13 | 5.48 | 1.97 | <20 | 28.13 | 388 | female | 52 | 114.98 | 136.14 | 25.38 |
| AGA-083 | 13.4 | 15.8 | 5.25 | 1.4 | 1.94 | 3.83 | 0.7 | <20 | 29.67 | 360 | male | 61 | 121.97 | 138.94 | 33.93 |
| AGA-084 | 8.57 | 20.66 | 8.42 | 1.31 | 2.45 | 3.84 | 0.99 | <20 | 26.41 | 423 | male | 46 | 87.02 | 120.54 | 36.18 |
| AGA-085 | 11.64 | 26.21 | 5.26 | 1.27 | 2.35 | 4.99 | 0.84 | <20 | 24.61 | 488 | male | 34 | 94.87 | 128.88 | 30.05 |
| AGA-086 | 8.24 | 13.15 | 5.2 | 1.23 | 4.52 | 6.71 | 1.89 | <20 | 29.86 | 316 | male | 51 | 103.06 | 139.48 | 25.16 |
| AGA-087 | 6.72 | 19.4 | 6.7 | 0.75 | 1.82 | 3.4 | 1.75 | <20 | 27.85 | 595 | male | 62 | 83.1 | 148.5 | 26.45 |
| AGA-088 | 9.2 | 18.23 | 4.19 | 0.98 | 3.19 | 4.7 | 1.38 | <20 | 27.18 | 450 | male | 38 | 88.82 | 146.67 | 33.13 |
| AGA-089 | 4.89 | 7.31 | 3.15 | 1.26 | 3.39 | 5.56 | 2.28 | <20 | 28.47 | 641 | male | 68 | 117.47 | 147 | 40.93 |
| AGA-090 | 4.67 | 15.83 | 6.85 | 1.08 | 2.89 | 4.73 | 2.6 | <20 | 29.92 | 521 | male | 91 | 106.56 | 139.42 | 22.5 |
| AGA-091 | 10.1 | 78.63 | 3.09 | 0.88 | 2.68 | 4.36 | 2 | <20 | 26.64 | 688 | male | 28 | 90.56 | 124.31 | 33.69 |
| AGA-092 | 10.18 | 4.4 | 2.98 | 1.05 | 2.81 | 4.39 | 1.68 | <20 | 31.61 | 479 | male | 32 | 127.39 | 127.63 | 27.82 |
| AGA-093 | 6.91 | 40.88 | 2.26 | 1.55 | 2.63 | 3.54 | 3.3 | <20 | 25.4 | 540 | male | 65 | 111.54 | 121.86 | 33.49 |
| AGA-094 | 9.12 | 5.14 | 2.09 | 1.45 | 2.54 | 5.17 | 1.6 | <20 | 23.16 | 354 | female | 59 | 71.77 | 121.29 | 34.81 |
| AGA-095 | 7.54 | 11.3 | 10.57 | 1.06 | 3.3 | 4.9 | 1.99 | <20 | 28.3 | 562 | male | 29 | 109.08 | 130.03 | 29.37 |
| AGA-096 | 8.15 | 71.4 | 2.8 | 1.37 | 1.09 | 3.12 | 1.38 | <20 | 25.89 | 350 | male | 57 | 89.24 | 136.59 | 45.21 |
| AGA-097 | 7.93 | 59.74 | 6.3 | 1.52 | 2 | 5.23 | 1.08 | <20 | 26.73 | 608 | male | 31 | 80.28 | 144.33 | 42.9 |
| AGA-098 | 5.9 | 17.76 | 3.31 | 0.76 | 3.18 | 5.55 | 0.61 | <20 | 27.35 | 537 | male | 46 | 79.26 | 142.28 | 15.1 |
| AGA-099 | 10.56 | 27.49 | 3.58 | 1.18 | 3.76 | 4.95 | 2.09 | <20 | 26.11 | 566 | male | 57 | 82.85 | 129.55 | 36.17 |
| **GIP group** | | | | | | | | | | | | | | | |
| GIP-075 | 6.2 | 1.2 | 6.95 | 1.03 | 4 | 2.43 | 1.02 | <20 | 24.48 | 405 | female | 51 | 94.36 | 146.24 | 15.94 |
| GIP-076 | 5.86 | 0.81 | 4.07 | 1.28 | 3.65 | 5.6 | 1.02 | <20 | 26.32 | 378 | male | 47 | 98.88 | 127.72 | 19 |
| GIP-077 | 9.57 | 10.76 | 7.25 | 1.24 | 2.87 | 5.74 | 2.59 | <20 | 25.77 | 419 | male | 40 | 104.93 | 125.58 | 18.76 |
| GIP-078 | 10.1 | 17.19 | 3.58 | 0.72 | 3.88 | 5.71 | 1.4 | <20 | 23.23 | 489 | male | 60 | 98.65 | 149.65 | 15.92 |
| GIP-079 | 8.31 | 6.5 | 7.16 | 0.78 | 2.65 | 4.94 | 2.29 | <20 | 24.54 | 435 | male | 48 | 94.8 | 141.54 | 17.5 |
| GIP-080 | 6.47 | 0.8 | 7.27 | 1.12 | 3.25 | 3.87 | 3.56 | <20 | 23.17 | 328 | male | 62 | 117.41 | 144.63 | 16.17 |
| GIP-081 | 10.02 | 39.73 | 6.33 | 0.86 | 2.4 | 3.7 | 1.14 | <20 | 25.52 | 491 | male | 41 | 115.34 | 134.96 | 18.16 |
| GIP-082 | 6.15 | 3.1 | 2.55 | 1.31 | 3.18 | 3.44 | 2.82 | <20 | 24.11 | 433 | male | 61 | 104.41 | 145.41 | 18.95 |
| GIP-083 | 5.58 | 1.3 | 2.89 | 1.35 | 2.01 | 8.2 | 1.3 | <20 | 25.62 | 393 | male | 45 | 92.64 | 127.07 | 15.4 |
| GIP-084 | 6.06 | 2.18 | 5.96 | 0.79 | 3.46 | 3.64 | 6.14 | <20 | 25.94 | 366 | male | 44 | 95.94 | 127.22 | 17.43 |
| GIP-085 | 10.02 | 28.09 | 7.09 | 1.06 | 3.52 | 5.98 | 2.7 | <20 | 26.86 | 479 | male | 65 | 90.37 | 121.85 | 14.6 |
| GIP-086 | 5.18 | 0.41 | 7.37 | 0.81 | 2.99 | 3.94 | 2.78 | <20 | 23.89 | 321 | male | 59 | 98.08 | 125.01 | 16.55 |
| GIP-087 | 6.62 | 7.33 | 7.14 | 1.15 | 3.89 | 4.18 | 2.04 | <20 | 26.84 | 347 | male | 41 | 96.19 | 127.65 | 14.82 |
| GIP-088 | 9.65 | 4.43 | 3.48 | 1.31 | 2.51 | 5.39 | 5.69 | <20 | 24.43 | 484 | male | 67 | 98.38 | 124.08 | 16.16 |
| GIP-089 | 10.72 | 27.79 | 4.87 | 0.99 | 2.48 | 3.85 | 6.08 | <20 | 24.81 | 384 | male | 44 | 95.11 | 136.12 | 14.1 |
| GIP-090 | 8.07 | 0.23 | 6.8 | 1.29 | 2.61 | 3.84 | 1.24 | <20 | 25.37 | 306 | male | 32 | 110.98 | 124.36 | 17.31 |
| GIP-091 | 6.8 | 0.87 | 3.77 | 0.94 | 3.89 | 6.8 | 1.92 | <20 | 26.87 | 315 | male | 60 | 107.17 | 142.99 | 15.73 |
| GIP-092 | 5.57 | 2.45 | 7.49 | 0.92 | 3.76 | 5.83 | 1.58 | <20 | 23.62 | 483 | male | 52 | 95.39 | 132.9 | 15.08 |
| GIP-093 | 4.88 | 0.85 | 6.12 | 0.86 | 3.54 | 3.89 | 1.78 | <20 | 24.71 | 452 | male | 55 | 95.26 | 129.05 | 15.34 |
| GIP-094 | 6.35 | 7.59 | 3.59 | 0.75 | 2.49 | 5.25 | 1.77 | <20 | 26.49 | 475 | female | 66 | 101.84 | 142.5 | 15.74 |
| GIP-095 | 4.89 | 10.56 | 1.72 | 1.11 | 3.1 | 5.89 | 3.3 | <20 | 25.8 | 332 | male | 43 | 111.91 | 129.29 | 15.38 |
| GIP-096 | 8.62 | 4.67 | 5.32 | 0.98 | 3.22 | 3.72 | 1.97 | <20 | 25.78 | 495 | male | 51 | 111.02 | 126 | 14.7 |
| GIP-097 | 9.53 | 1.63 | 3.64 | 0.97 | 2.16 | 5.01 | 6.67 | <20 | 23.58 | 439 | male | 40 | 109.34 | 134.83 | 14.88 |
| GIP-098 | 9.52 | 7.25 | 4.71 | 1.3 | 2.2 | 5.8 | 5.47 | <20 | 25.94 | 315 | male | 37 | 115.74 | 141.01 | 15.21 |
| GIP-099 | 6.46 | 6.41 | 5.83 | 0.99 | 3.39 | 4.51 | 1.32 | <20 | 24.23 | 386 | male | 69 | 92.73 | 137.33 | 18.49 |
| GIP-100 | 5.7 | 3.87 | 3.64 | 1.4 | 3.14 | 5.67 | 1.02 | <20 | 26.86 | 348 | male | 64 | 113.63 | 138.97 | 14.37 |
| GIP-101 | 8.07 | 4.2 | 4.37 | 1.21 | 3.68 | 4.13 | 2.32 | <20 | 23.34 | 357 | male | 55 | 106.28 | 148.3 | 14.56 |
| GIP-102 | 7.53 | 8.43 | 5.79 | 1.26 | 2.87 | 3.6 | 2.38 | <20 | 23.73 | 373 | male | 35 | 108.93 | 140.98 | 14.49 |
| GIP-103 | 5.37 | 1.78 | 3.61 | 1.39 | 3.74 | 5.58 | 1.25 | <20 | 25.27 | 442 | male | 37 | 103.94 | 147.65 | 16.32 |
| GIP-104 | 6.98 | 2.12 | 7.14 | 0.97 | 2.77 | 7.94 | 1.69 | <20 | 23.09 | 360 | male | 31 | 116.99 | 127.24 | 18.13 |
| **CGA group** | | | | | | | | | | | | | | | |
| CGA-063 | 12.26 | 23.36 | 10.6 | 1.08 | 3.3 | 4.35 | 2.23 | <20 | 20.44 | 354 | male | 65 | 216.34 | 130.29 | 22.52 |
| CGA-064 | 6.92 | 3.94 | 11.86 | 1.64 | 3.49 | 4.71 | 3.38 | <20 | 22.31 | 450 | male | 80 | 165.05 | 149.23 | 25.33 |
| CGA-065 | 5.13 | 1.86 | 11.09 | 0.85 | 2.63 | 7.73 | 1.91 | <20 | 21.65 | 438 | male | 63 | 226.08 | 143.75 | 19.72 |
| CGA-066 | 6.29 | 7.14 | 7.92 | 1.52 | 2.15 | 4.74 | 1.88 | <20 | 20.17 | 314 | male | 66 | 154.74 | 141.8 | 27.58 |
| CGA-067 | 9.87 | 4.22 | 7.25 | 1.43 | 3.98 | 5.48 | 3.56 | <20 | 22.52 | 386 | male | 65 | 245.31 | 137.52 | 28.16 |
| CGA-068 | 7.17 | 2.82 | 7.04 | 1 | 1.77 | 5.32 | 2.11 | <20 | 19.19 | 446 | male | 78 | 175.22 | 145.32 | 21.72 |
| CGA-069 | 10.87 | 16.54 | 6.44 | 1.14 | 2.68 | 3.86 | 3.32 | <20 | 19.78 | 346 | male | 72 | 173.64 | 147.36 | 26.4 |
| CGA-070 | 4.11 | 1.26 | 11.62 | 1.32 | 3.95 | 6.42 | 2.59 | <20 | 22.69 | 431 | male | 71 | 236.3 | 143.05 | 22.63 |
| CGA-071 | 4.95 | 14.92 | 10.2 | 1.13 | 2.86 | 5.24 | 2.38 | <20 | 22.01 | 353 | male | 87 | 168.99 | 140.78 | 30.96 |
| CGA-072 | 6.53 | 6.08 | 11.27 | 1.06 | 3.29 | 5.81 | 1.28 | <20 | 20.32 | 262 | male | 69 | 222.29 | 130.98 | 27.61 |
| CGA-073 | 6.37 | 8.66 | 10.64 | 1.37 | 1.83 | 5.01 | 1.56 | <20 | 19.7 | 428 | male | 74 | 248.56 | 135.69 | 25.21 |
| CGA-074 | 5.03 | 3.85 | 9.91 | 1.59 | 4.07 | 5.59 | 2.4 | <20 | 21.65 | 302 | male | 64 | 217.13 | 124.79 | 23.75 |
| CGA-075 | 5.01 | 27.81 | 8.25 | 1.25 | 2.39 | 4.77 | 2.07 | <20 | 19.11 | 278 | male | 82 | 214.1 | 147.64 | 33.66 |
| CGA-076 | 6.47 | 28.2 | 7.81 | 1.53 | 4.01 | 3.82 | 3.86 | <20 | 21.77 | 267 | male | 77 | 151.39 | 134.69 | 19.37 |
| CGA-077 | 10.01 | 11.32 | 8.03 | 1.39 | 2.93 | 5.43 | 1.39 | <20 | 25.87 | 351 | male | 78 | 228.15 | 138.31 | 33.29 |
| CGA-078 | 4.76 | 8.76 | 9.41 | 1.04 | 3.31 | 6.98 | 2.37 | <20 | 22.56 | 294 | male | 81 | 237.46 | 127.57 | 25.39 |
| CGA-079 | 5.7 | 5.78 | 11.39 | 1.4 | 2.87 | 4.38 | 3.05 | <20 | 26.46 | 363 | male | 85 | 179.95 | 142.63 | 27.35 |
| CGA-080 | 11.68 | 20.46 | 9.01 | 1.51 | 2.1 | 4.77 | 3.55 | <20 | 23.21 | 397 | male | 76 | 209.17 | 147.3 | 26.74 |
| CGA-081 | 9.85 | 24.61 | 8.11 | 1.41 | 3.67 | 4.21 | 3.72 | <20 | 23.31 | 427 | male | 73 | 169.39 | 128.48 | 17.03 |
| CGA-082 | 7.02 | 3.56 | 10.18 | 0.93 | 2.87 | 5.24 | 1.8 | <20 | 21.04 | 261 | male | 84 | 229.88 | 132.17 | 17.2 |
| CGA-083 | 10.74 | 19.3 | 6.86 | 1.26 | 4.23 | 4.04 | 1.73 | <20 | 23.44 | 246 | male | 70 | 211.44 | 130.91 | 20.97 |
| CGA-084 | 9.01 | 3.49 | 7.69 | 1.55 | 1.75 | 6.45 | 2.11 | <20 | 26.26 | 236 | male | 64 | 222.36 | 142.9 | 26.19 |
| CGA-085 | 6.2 | 5.9 | 9.61 | 1.27 | 3.73 | 4.07 | 1.3 | <20 | 23.88 | 331 | male | 77 | 150.69 | 126.11 | 23.32 |
| CGA-086 | 5.17 | 22.59 | 6.96 | 1.54 | 3.98 | 4.16 | 3.74 | <20 | 21.11 | 450 | male | 84 | 207.7 | 135.81 | 29.85 |
| CGA-087 | 7.6 | 20.79 | 9.51 | 0.82 | 4.1 | 5.44 | 2.33 | <20 | 23.94 | 359 | male | 65 | 242.7 | 134.77 | 23.54 |
| CGA-088 | 8.36 | 10.81 | 6.9 | 1.41 | 2.24 | 5.63 | 1.73 | <20 | 19.01 | 324 | male | 85 | 222.26 | 131.17 | 23.72 |
| CGA-089 | 9.63 | 4.81 | 9.13 | 0.81 | 4.17 | 4.66 | 0.82 | <20 | 19.19 | 320 | male | 64 | 194.15 | 121.79 | 23.31 |
| CGA-090 | 9.25 | 7.74 | 9.5 | 1.27 | 2.23 | 3.83 | 3.08 | <20 | 25.72 | 429 | male | 65 | 236.57 | 128.73 | 30.61 |
| CGA-091 | 6.78 | 12.3 | 7.88 | 1.55 | 2.94 | 3.64 | 1.13 | <20 | 20.35 | 409 | male | 70 | 198.26 | 149.76 | 23.92 |
| CGA-092 | 10.03 | 15.53 | 6.39 | 1.38 | 2.03 | 7.35 | 1.17 | <20 | 22.87 | 231 | male | 87 | 196.32 | 133.07 | 17.46 |
